# Impact of cannabis use on brain metabolism using ^31^P and ^1^H magnetic resonance spectroscopy

**DOI:** 10.1007/s00234-023-03220-y

**Published:** 2023-09-22

**Authors:** Maximilian Fenzl, Martin Backens, Silviu Bodea, Miriam Wittemann, Florian Werler, Jule Brielmaier, Robert Christian Wolf, Wolfgang Reith

**Affiliations:** 1 Institute of Neuroradiology, Saarland University, 66421 Homburg, Germany; 2 Helmholtz Zentrum Munich, German Research Center for Environmental Health Institute of Biological and Medical Imaging, 85748 Munich, Germany; 3 Department of Psychiatry and Psychotherapy, Saarland University, 66421 Homburg, Germany; 4 Department of General Psychiatry at the Center for Psychosocial Medicine, Heidelberg University, 69115 Heidelberg, Germany; 5 Department of Obstetrics and Gynecology, RKH Clinic Ludwigsburg, 71640 Ludwigsburg, Germany

**Keywords:** Cannabis, Marijuana, 1H MRS, 31P MRS, Brain metabolites

## Abstract

**Purpose:**

This prospective cross-sectional study investigated the influence of regular cannabis use on brain metabolism in young cannabis users by using combined proton and phosphorus magnetic resonance spectroscopy.

**Methods:**

The study was performed in 45 young cannabis users aged 18–30, who had been using cannabis on a regular basis over a period of at least 2 years and in 47 age-matched controls. We acquired 31P MRS data in different brain regions at 3T with a double-resonant 1H/31P head coil, anatomic images, and 1H MRS data with a standard 20-channel 1H head coil. Absolute concentration values of proton metabolites were obtained via calibration from tissue water as an internal reference, whereas a standard solution of 75 mmol/l KH2PO4 was used as an external reference for the calibration of phosphorus signals.

**Results:**

We found an overall but not statistically significant lower concentration level of several proton and phosphorus metabolites in cannabis users compared to non-users. In particular, energy-related phosphates such as adenosine triphosphate (ATP) and inorganic phosphate (Pi) were reduced in all regions under investigation. Phosphocreatine (PCr) showed lowered values mainly in the left basal ganglia and the left frontal white matter.

**Conclusion:**

The results suggest that the increased risk of functional brain disorders observed in long-term cannabis users could be caused by an impairment of the energy metabolism of the brain, but this needs to be verified in future studies.

**Supplementary Information:**

The online version contains supplementary material available at 10.1007/s00234-023-03220-y.

## Introduction

Cannabis is one of the most widely used recreational drugs in the world [1]. Even though there has been a concern over decades about the use of cannabis as a cause of psychiatric illness, cannabis-related disorders have been rising among the past years [2]. Partial legalization can be associated with the increasing usage and the reduction in the perception of harm [3]. Due to this development, more scientific evidence is needed to determine the degree of harmfulness to the human body, especially with respect to brain metabolism and the whole nervous system.

Delta-9-tetrahydrocannabinol (∆9-THC) is the main psychoactive component of cannabis, acting on cannabinoid (CB1) receptors which can be densely found within brain networks critical for learning, attention, memory, cognitive processing, and motor control [4]. Moderate to high concentrations of CB1-binding sites have been detected in the thalamus, cerebellum, amygdala, basal ganglia, occipito-temporal gyrus, inferior temporal gyrus, frontal cortex, and hippocampus [4–6].

Several studies have shown that long-term cannabis use negatively affects memory, motor skills, executive function, emotional processing, and attention in adolescents [7–9] and adults [10–14]. In neuroimaging studies, long-term cannabis users exhibited abnormal brain activation during performance of functional tasks, including decision-making, verbal list learning, visual attention, and response inhibition [15–18].

Proton MRS is a non-invasive technique that has been widely applied to detect and quantify important neurometabolites [19]. Using single-voxel or multi-voxel acquisition schemes, cerebral metabolites including NAA (N-acetyl-aspartate), Cr (creatine), and cytosolic choline (Cho) can be assessed. NAA plays a role as a biomarker indicating neuronal viability [20]. Total Cr (tCr, creatine plus phosphocreatine) is involved in the energy metabolism, acting as an energy buffer by distributing energy within the brain and by maintaining constant brain adenosine triphosphate (ATP) levels through the creatine kinase reaction [21, 22]. The Cho signal is associated with cellular membrane synthesis and degradation.

Phosphorus MRS in addition allows in vivo evaluation of compounds directly related to the energy metabolism and the composition of cell membranes. Adenosine triphosphate (ATP), phosphocreatine (PCr), and inorganic phosphate (Pi) are linked to brain bioenergetics through biochemical energy production (i.e., ATP synthesis) and energy use (i.e., ATP utilization). The phosphomonoesters (PME) play an important role in the synthesis of membrane lipids such as phosphatidylcholine and phosphatidylethanolamine. The main PME constituents, phosphoethanolamine (PE) and phosphocholine (PC), are precursors of the corresponding phospholipids. Membrane breakdown, in turn, is indicated by the phosphodiester (PDE) and catabolic products of phospholipid metabolism, glycerol-phosphoethanolamine (GPE), and glycerol-phosphocholine (GPC). Decreased membrane turnover has been associated with elevated PDE levels [23]. PME reduction refers to altered membrane turnover rates. In bipolar depression, e.g., studies have shown significantly altered frontal lobe PME [24]. Furthermore, 31P MRS can detect nicotinamide adenine dinucleotide phosphate (NADP), which is involved in oxidative chains and in membrane phospholipid metabolism [25]. Finally, it is possible to obtain the value of intracellular pH as well as the concentration of magnesium (Mg2+) from the spectrum [26, 27].

To the best of our knowledge, no literature is available on brain metabolic changes using 31P-MRS related to cannabis use. In this study, we used both single-voxel-1H MRS and multi-voxel-31P MRS at a 3 T scanner to determine absolute metabolite concentration values from five brain areas that are suspected to be affected by cannabis [28] including frontal gray (FGM) and frontal white matter (FWM), thalamus (TH), basal ganglia (BG), and temporal lobe (TL). With 31P MRS, all regions except FGM were evaluated in both hemispheres separately. 1H MRS voxels other than FGM were restricted to the right hemisphere. Comparing concentration data between long-term cannabis users and non-users, we detected considerable though not statistically significant differences which might help to better understand the impact of cannabis use on brain metabolism. In addition, sex-related differences in non-users were found.

## Methods

### Study subjects

The subjects were recruited through local drug counseling centers. None of the participants received treatment for substance-use disorder. Control subjects were recruited through advertisement (poster, flyer) at the hospital. Recruitment of both subject groups took place simultaneously. All subjects were interviewed by an experienced psychologists or psychiatrist to assess extent and history of their cannabis use and underwent a complex psychometric assessment (supplementary_Demographics: suppl_table 1) to ensure inclusion criteria as seen below [29].

Only right-handed study subjects and controls between 18 and 30 years without neurological, psychiatric, and systemic diseases and without further drug addictions were included. This restriction was meant to exclude the effects of handedness and medical conditions on brain metabolism.

We investigated 21 female non-users (fN) (age 23 ± 2) and 26 male non-users (mN) (age 25 ± 4), who had never been using cannabis before or less than 10 times in total (= lifetime consume).

In the consumer group, 5 female cannabis-users (fC) (age 24 ± 4) and 40 male cannabis-users (mC) (age 24 ± 3) were examined. All users had been using cannabis on a regular basis at least 1 day per month in the last 24 months.

The fC group was excluded from further evaluation because not sufficient subjects could be found during the study.

Before MRI scan, study participants had to remove all metal objects. The subjects were instructed to move as little as possible during the MR examination which lasted about 1.5 h. Smoking was prohibited on examination day.

In this study, we used the STROBE cross-sectional reporting guidelines [30].

### Data acquisition

Data acquisition of the brain was performed on a 3T whole body system (Magnetom Skyra, Siemens Healthcare, Erlangen, Germany). For anatomic images and 1H MRS, the standard 20-channel 1H (receive-only) head coil was used due to quality reasons. 31P spectra were acquired using a double-resonant 1H/31P (transmit/receive) head coil (RAPID Biomedical GmbH, Rimpar, Germany).

Anatomical data included three orthogonal T2-weighted localizers and a sagittal 3D T1-weighted data set (resolution 0.9 mm × 0.9 mm × 0.9 mm) of the whole brain (MPRAGE) which allowed segmentation of the brain tissue to obtain compartment maps of gray matter, white matter, and CSF. Segmentation was obtained using the SPM software (SPM 8, statistical parametric mapping, The Wellcome Trust Centre for Neuroimaging, University College London). For all spectroscopic volumes of interest, volume fractions of the three compartments were calculated from the maps.

Single-voxel proton spectra were obtained from 4 different brain regions: frontal gray matter (FGM), right frontal white matter (r_FWM), right thalamus (r_TH), and right temporal region (r_TL). Because of time restrictions, no spectra were acquired from the left hemisphere. Mean voxel size was 15 ml, 12 ml, 10 ml, and 8 ml respectively. Depending on brain size, voxel size was individually slightly adjusted to ensure accurate coverage of the anatomical target region. We used a PRESS sequence with TR = 1500 ms, TE = 135 ms, 80 acquisitions, bandwidth = 1200 Hz, and vector size = 1024. Shim adjustment was corrected manually to achieve minimal line width. As tissue water was used as an internal reference for absolute quantification of metabolites, additional spectra without water suppression were acquired from each voxel.

After coil change and repositioning of the patient phosphorus spectra were recorded using a 3D-chemical-shift-imaging (CSI) free-induction-decay (FID) sequence (TR = 1200 ms, TE = 2.3 ms, 15 acquisitions, bandwidth = 2000 Hz, vector size = 1024). Elliptical phase encoding with a weighted acquisition scheme was employed. Matrix size was 8 × 8 × 8, FOV = 200 × 200 mm^2^ resulting in 25 × 25 × 25 mm^3^ voxels. Optimized signal intensity was achieved by applying proton decoupling using the WALTZ-4 scheme and by a reduced flip angle of 60°. Careful manual shimming of the 3D volume was applied yielding line widths lower than 30 Hz. Acquisition time was 8:42 min. For absolute quantification of phosphorus metabolites, a phosphorus phantom with 75 mmol/l KH2PO4 was used as an external reference. The phantom was placed in the headcoil close to the left fronto-parietal part of the head.

Further information concerning data quality of 31P MRS and 1H MRS spectra can be found in supplementary_P_DataQuality and supplementary_H_DataQuality, respectively.

### Data processing

Evaluation of proton spectra was done using the commercial software tool LCModel [31] (http://s-provencher.com/lcmodel.shtml). The signal-to-noise ratio (SNR) and the value of Cramer-Rao lower bound (%SD) were used to discard low quality data. Only spectra with SNR higher than 3 and %SD lower than 20% both for Cr and Cho were included for further analysis. To obtain absolute metabolite concentration values, the LCModel output data were corrected for longitudinal and transversal relaxation of both metabolites and brain tissue water taking into account the fractions of GM, WM, and CSF determined separately for each voxel from the segmentation maps. Relative tissue water content of 78%, 65%, and 97% was assumed for GM, WM, and CSF, respectively [32]. Relaxation factors (*R*_H_) were calculated according to the following equation for double-spin-echo sequences [33]:

$${S}_H={S}_{H0}\cdot {\mathrm{R}}_H$$

with

$${R}_H=\exp \left(-\frac{TE}{T2}\right)\left\{1-\exp \left(-\frac{TR}{T1}\right)+2\exp \left(\left[\left(\frac{TE}{2}\right)- TR\right]/T1\right)-2\exp \left(\left[\left(\frac{3\ TE}{2}\right)- TR\right]/T1\right)\right\}$$

*S*_*H*_ and *S*_*H*0_ represent the measured proton peak intensity and the peak intensity corrected for T1 and T2 relaxation, respectively. T1 and T2 values were chosen as mean values (supplementary_H_Results: suppl_table 6) from the literature [34–47]. Finally, concentrations in units of milli-mole (mMol) per kg of brain tissue were calculated by correcting all metabolite values for the CSF fraction of each spectroscopic voxel determined from the compartment maps.

**Fig. 1:**
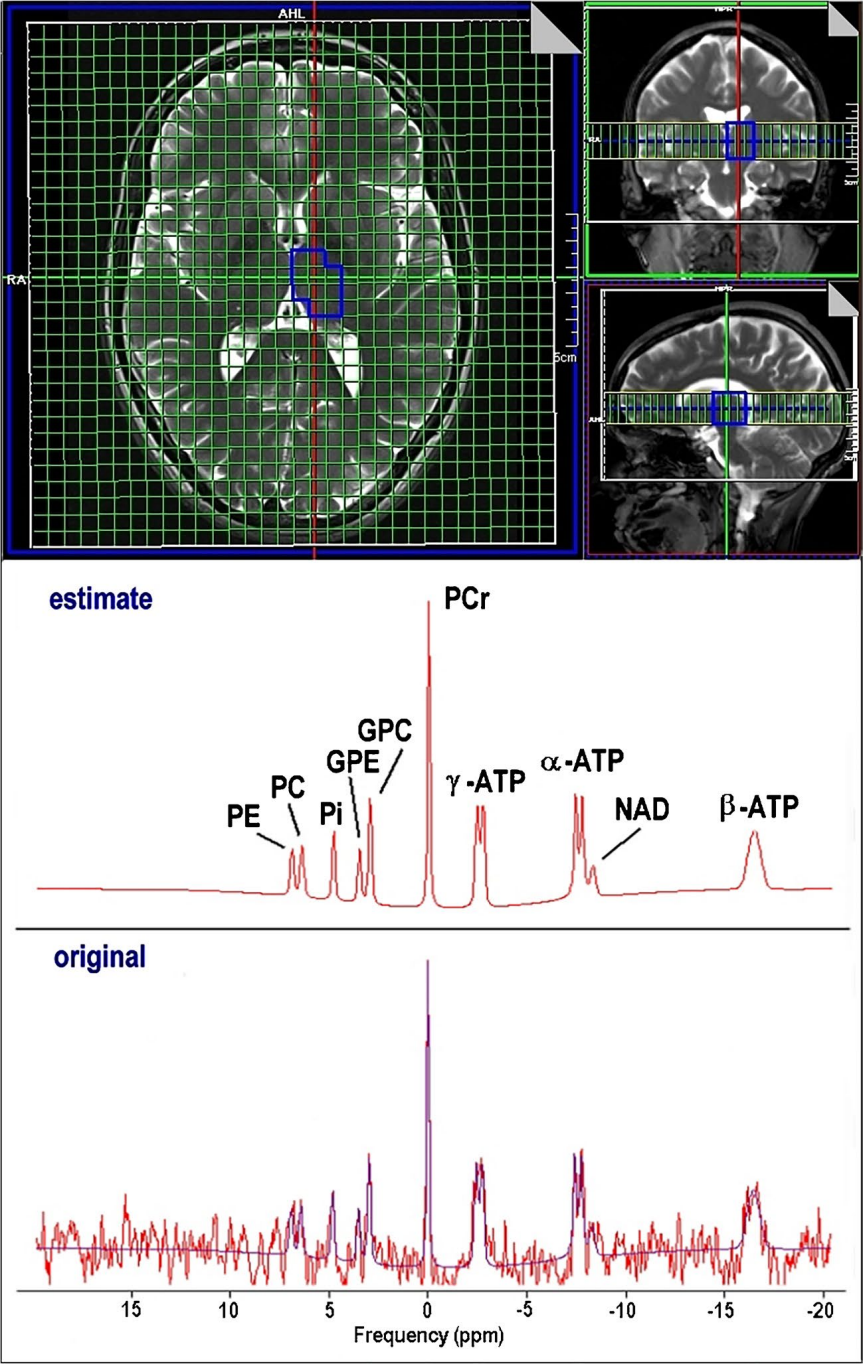
Selection of the anatomical region of interest for 31P spectroscopic evaluation with the scanner software (Siemens Leonardo workstation) and final jMRUI results after processing AMARES algorithm

Phosphorus CSI data were transferred to a Leonardo workstation (Siemens Healthcare GmbH, Erlangen, Germany) and interpolated to a 32 × 32 × 8 grid resulting in a stack of 8 axial slices with 25 mm thickness (voxel size 6.3 × 6.3 × 25 mm^3^ ≈ 1 ml) which were superimposed on axial T2-weighted slices (Fig. 1). Anatomical volumes of interest (VOI) for spectral evaluation were identified by manually (M.F., M.B., and S.B.) selecting appropriate voxels in the grid. Grid shift in-plane as well as in head-feet direction was applied to optimally enclose the respective anatomical region of interest. Nine different VOIs were delineated for each subject in FGM, l_FWM, r_FWM, l_TH, r_TH, l_BG, r_BG, l_TL, and r_TL. VOI size ranged from 7 to 18 ml.

Quantitative analysis of the 31P spectra was performed with the jMRUI software tool (version 5.1) employing the AMARES algorithm [48]. The model function was composed of 14 resonances including PE, PC, Pi, GPE, GPC, PCr, ATP, and one macromolecular component to account for the broad signal baseline (Fig. 1). ATP was represented by a total of 7 peaks: a doublet γ-ATP, a doublet α-ATP, and a triplet β-ATP. Constraints for frequency, damping, coupling constants, and amplitude ratios (prior knowledge) were defined for the compounds to be estimated by the algorithm. The resulting amplitude values are proportional to the corresponding metabolite concentration. The concentration of ATP was calculated from the γ-ATP resonance. Only spectra with SNR higher than 3 were included for further analysis.

The AMARES algorithm provides Cramer-Rao lower bound (sd.amp.) values as an error estimate for all peaks in each spectrum. Whereas PCr signals always had relative error values lower than 20%, weak signals, e.g., NAD and PC, suffer from low intensities and high errors. Peaks with relative error values > 1 were excluded from further analysis.

Several postprocessing steps are required to obtain absolute quantification of metabolites: first, the signal amplitudes were corrected for the reduced flip angle and for T1 relaxation. Correction factors (*R*_*P*_) were calculated using the following equation:

$${S}_P={S}_{P0}\cdot {R}_P$$

with

$${R}_P=\frac{\sin (x)\left\{\ 1-\exp \left(-\frac{TR}{T1}\right)\ \right\}}{\left\{\ 1-\cos (x)\exp \left(-\frac{TR}{T1}\right)\right\}},\kern0.5em x=60{}^{\circ},\mathrm{TR}=1200\ \mathrm{ms}.$$

*S*_*P*_ and *S*_*P*0_ represent the measured phosphorus peak intensity and the peak intensity corrected for T1 relaxation and flip angle, respectively (supplementary_P_Results, suppl_table 4).

Varying coil loading due to different head sizes of subjects was taken into account based on the radiofrequency transmitter amplitude required for a 90° pulse. Calibration of signal intensities was done with the phantom replacement method [49]. Finally, the calculated metabolic concentrations were corrected for partial CSF volume of each VOI to obtain concentration values in units of mMol per kg of brain tissue.

### Calculated parameters

Intracellular pH was calculated from the chemical shift difference *δ* between the peak of inorganic phosphate (Pi) and the PCr peak [50–52] according to the equation:

$$\mathrm{pH}=6.75+{\log}_{10}\left[\frac{3.27-\updelta}{\updelta -5.63}\right]$$

Free cytosolic Mg^2+^ was estimated from the chemical shift difference *δ*_β_ between the peak of β-ATP and the PCr peak according to the formula:

$$\mathrm{pMg}=4.24-{\log}_{10}\left[\frac{{\left({\delta}_{\upbeta}+18.58\right)}^{0.42}}{{\left(-15.74-{\delta}_{\upbeta}\right)}^{0.84}}\right]$$

The relation between the concentration of Mg^2+^ (in mol/l) and the value of pMg is given by: [Mg^2+^] =  − log_10_(pMg).

Concentration ratios of PCr and Cr were estimated for those ROIs, where both phosphorus and proton spectra were acquired: FWM, r_TH, r_TL, and r_FWM. Cr values were calculated as [Cr] = [tCr] − [PCr].

### Statistical methods

All statistical evaluations were performed using IBM SPSS Statistics (Version 27). Mean values for each metabolite concentration as well as for pH and Mg were calculated for every VOI separately. Concentration differences between groups were determined as relative values in percent according to:

$$\Delta \mathrm{mf}=\frac{\left(\mathrm{mN}-\mathrm{fN}\right)}{\mathrm{fN}}, \Delta \mathrm{CN}=\frac{\left(\mathrm{mC}-\mathrm{mN}\right)}{\mathrm{mN}}$$

The statistical analysis was based on the General Linear Model using multivariate analysis of variance (MANOVA).

For P MRS, the metabolite values of PME, Pi, PDE, PCr, ATP, pH, and Mg were set as dependent variables, while membership to one of the three groups (fN, mN, and mC) was set as a fixed factor. NAD was excluded from the analysis because of too many low-quality data. To investigate the overall effect of the groups on all seven metabolite values, a multivariate Wilks-Lambda test was used. Paired comparisons were performed by post hoc Scheffé test. The level of significance was corrected for multiple tests using the Bonferroni approach. We analyzed nine regions simultaneously, so a *p* < 0.0056 was chosen as the criterion for significance.

Side related differences in metabolite values were calculated as relative values in percent according to:

$$\Delta \mathrm{rl}=\frac{\left(\mathrm{right}-\mathrm{left}\right)}{\mathrm{left}}.$$

For statistical evaluation, multivariate analysis of variance with repeated measurements (RM MANOVA) was used in four regions: TH, BG, TL, and FWM. To investigate overall hemispheric effects, the metabolite values from the left and right hemisphere were set as within-subject factors, membership to the groups was set as a between-subjects factor. As we made four bilateral comparisons, the level of significance was chosen as *p* < 0.0125 according to the Bonferroni approach. In order to compare side related effects between the groups, additional RM ANOVAs were performed for each group separately. Hemispheric differences for individual metabolite values were evaluated with paired *t* test.

For H MRS, the metabolic values of tNAA, tCr, and tCho were set as dependent variables in the MANOVA. To investigate the overall effect of the groups on all three metabolite values, a multivariate Wilks-Lambda test was used. Paired comparisons were performed by post hoc Scheffé test. The level of significance was corrected for multiple tests using the Bonferroni approach. We measured four regions, so a *p* < 0.0125 was chosen as the criterion for significance.

## Results

**Fig. 2:**
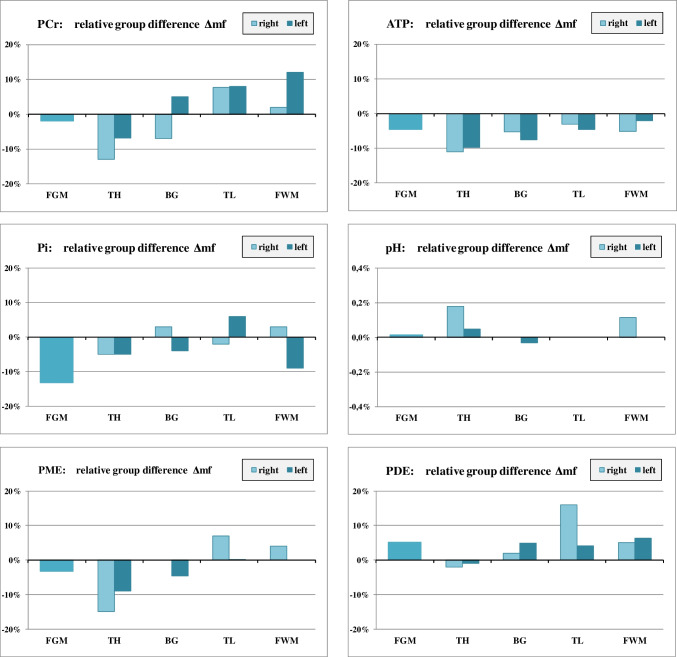
Results of 31P MRS: relative group difference of metabolite concentrations between male (mN) and female (fN) non-consumers: $\Delta \mathrm{mf}=\frac{\left(\mathrm{mN}-\mathrm{fN}\right)}{\mathrm{fN}}$. Regional variation of Δmf for selected metabolites

**Fig. 3:**
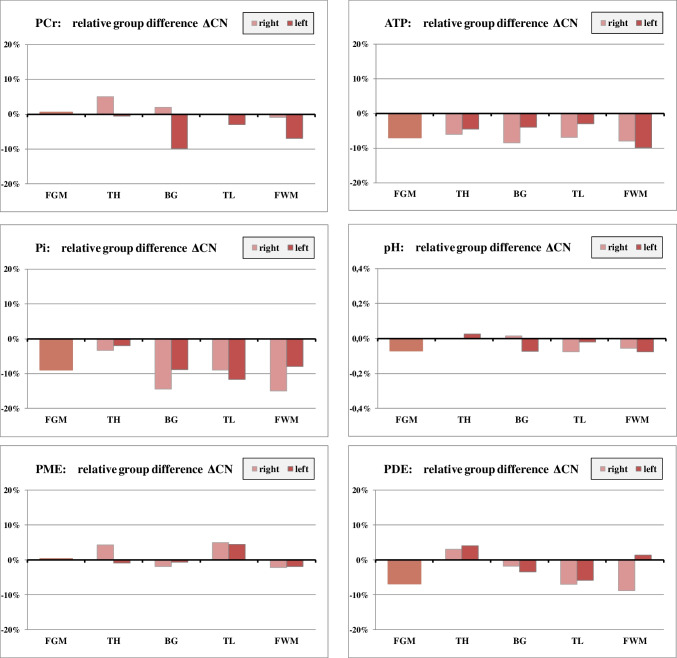
Results of 31P MRS: relative group difference of metabolite concentrations between male cannabis-consumers (mC) and male non-consumers (mN): $\Delta \mathrm{CN}=\frac{\left(\mathrm{mC}-\mathrm{mN}\right)}{\mathrm{mN}}$. Regional variation of ΔCN for selected metabolites

**Fig. 4:**
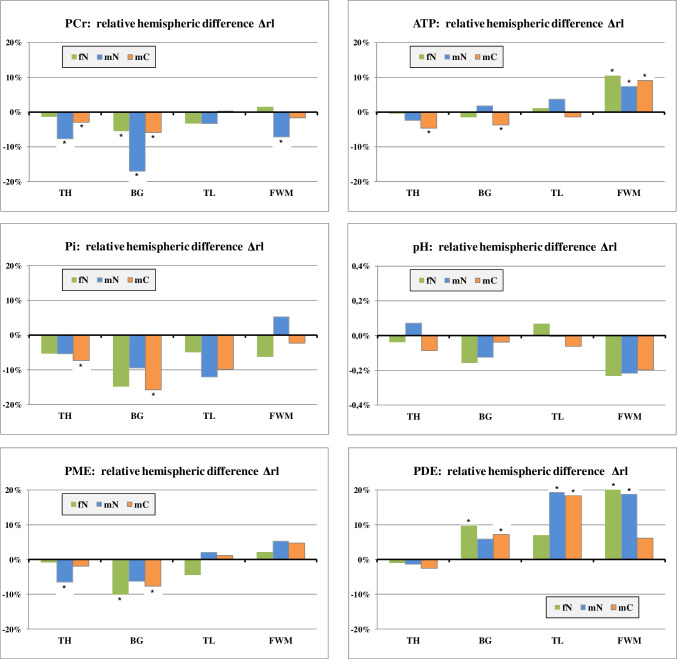
Results of 31P MRS: relative hemispheric difference Δrl of metabolic values for fN, mN, and mC: $\Delta \mathrm{rl}=\frac{\left(\mathrm{right}-\mathrm{left}\right)}{\mathrm{left}}$. Asterisk marks *p* values < 0.05

**Fig. 5:**
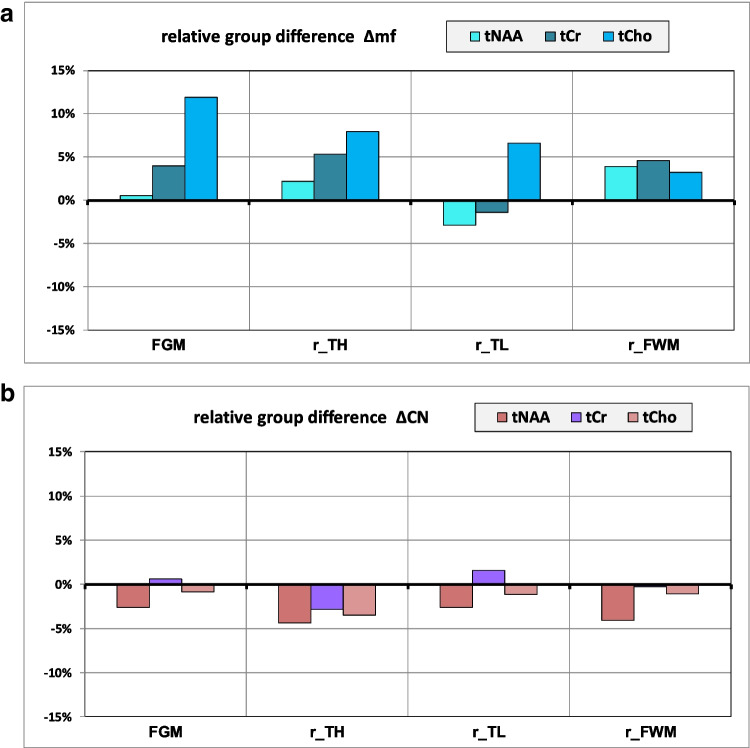
**a** Relative group difference of metabolite concentrations between male (mN) and female non-consumers (fN): $\Delta \mathrm{mf}=\frac{\left(\mathrm{mN}-\mathrm{fN}\right)}{\mathrm{fN}}$. **b** Relative group difference of metabolite concentrations between male cannabis-consumers (mC) and male non-consumers (mN): $\Delta \mathrm{CN}=\frac{\left(\mathrm{mC}-\mathrm{mN}\right)}{\mathrm{mN}}$

31P MRS results are shown in Tables 1 and 2 and Figs. 2, 3, and 4; more detailed data can be found in supplementary_P_results (suppl_table 5 and suppl_figs. 9a–d). 1H MRS results are shown in Table 3 and Fig. 5; more detailed data are given in supplementary_H_results (suppl_table 7, suppl_table 8 and suppl_figs.10a–b).

**Table 1:** Results of 31P MRS, comparison between groups

FGM	MANOVA: Wilks-Lambda: *p* = 0.175				
		Post hoc Scheffé test **ΔCN**			
	**fN**	**mN**	**mC**	rel. diff.	*p* value
PME	2.70	2.61	2.62	0%	0.997
Pi	0.68	0.59	0.54	− 9%	0.599
PDE	2.89	3.04	2.83	− 7%	0.259
PCr	3.65	3.58	3.60	1%	0.984
ATP	2.83	2.70	2.51	− 7%	0.400
pH	6.98	6.99	6.98	− 0.1%	0.343
Mg	0.11	0.10	0.10	1%	0.923
r_TH	MANOVA: Wilks-Lambda: *p* = **0.014**				
		Post hoc Scheffé test **ΔCN**			
	**fN**	**mN**	**mC**	rel. diff.	*p* value
PME	2.30	1.96	2.05	4%	0.680
Pi	0.79	0.75	0.73	− 3%	0.892
PDE	2.61	2.56	2.63	3%	0.790
PCr	3.70	3.23	3.38	5%	0.558
ATP	2.43	2.16	2.03	− 6%	0.586
pH	6.99	6.99	6.99	0.0%	0.827
Mg	0.10	0.11	0.10	− 5%	0.469
r_BG	MANOVA: Wilks-Lambda: *p* = 0.358				
		Post hoc Scheffé test **ΔCN**			
	**fN**	**mN**	**mC**	rel. diff.	*p* value
PME	2.17	2.16	2.11	− 2%	0.874
Pi	0.61	0.63	0.53	− 15%	0.117
PDE	2.66	2.70	2.63	− 2%	0.846
PCr	3.48	3.22	3.29	2%	0.865
ATP	2.49	2.36	2.15	− 9%	0.233
pH	6.99	6.99	6.99	0.0%	0.998
Mg	0.11	0.11	0.11	0%	0.991
r_TL	MANOVA: Wilks-Lambda: *p* = **0.034**				
		Post hoc Scheffé test **ΔCN**			
	**fN**	**mN**	**mC**	rel. diff.	*p* value
PME	2.03	2.18	2.25	4%	0.764
Pi	0.59	0.58	0.53	− 9%	0.453
PDE	2.03	2.36	2.20	− 7%	0.487
PCr	3.50	3.78	3.79	0%	1.000
ATP	2.30	2.23	2.07	− 7%	0.367
pH	7.00	7.00	6.99	− 0.1%	0420
Mg	0.12	0.11	0.11	− 2%	0.854
r_FWM	MANOVA: Wilks-Lambda: *p* = 0.216				
		Post hoc Scheffé test **ΔCN**			
	**fN**	**mN**	**mC**	rel. diff.	*p* value
PME	2.27	2.35	2.30	− 2%	0.894
Pi	0.61	0.63	0.54	− 15%	0.145
PDE	2.56	2.69	2.44	− 9%	0.118
PCr	3.40	3.46	3.42	− 1%	0.941
ATP	2.60	2.47	2.27	− 8%	0.199
pH	6.98	6.99	6.98	− 0.1%	0.738
Mg	0.11	0.11	0.11	1%	0.970
l_TH	MANOVA: Wilks-Lambda: *p* = 0.359				
		Post hoc Scheffé test **ΔCN**			
	**fN**	**mN**	**mC**	rel. diff.	*p* value
PME	2.32	2.10	2.09	− 1%	0.986
Pi	0.84	0.80	0.78	− 2%	0.976
PDE	2.63	2.60	2.70	4%	0.943
PCr	3.75	3.50	3.48	− 1%	0.996
ATP	2.44	2.21	2.12	− 4%	0.773
pH	6.99	6.99	6.99	0.0%	0.992
Mg	0.10	0.11	0.10	− 6%	0.146
l_BG	MANOVA: Wilks-Lambda: *p* = 0.122				
		Post hoc Scheffé test **ΔCN**			
	**fN**	**mN**	**mC**	rel. diff.	*p* value
PME	2.41	2.30	2.29	− 1%	0.985
Pi	0.72	0.69	0.63	− 9%	0.488
PDE	2.42	2.54	2.46	− 3%	0.698
PCr	3.68	3.88	3.50	− 10%	**0.015**
ATP	2.53	2.32	2.23	− 4%	0.766
pH	7.00	7.00	6.99	− 0.1%	0.710
Mg	0.11	0.11	0.11	0%	0.995
l_TL	MANOVA: Wilks-Lambda: *p* = 0.579				
		Post hoc Scheffé test **ΔCN**			
	**fN**	**mN**	**mC**	rel. diff.	*p* value
PME	2.13	2.13	2.23	4%	0.631
Pi	0.63	0.67	0.59	− 12%	0.366
PDE	1.90	1.98	1.86	− 6%	0.518
PCr	3.62	3.91	3.78	− 3%	0.792
ATP	2.27	2.15	2.10	− 3%	0.870
pH	7.00	7.00	7.00	0.0%	0.987
Mg	0.11	0.11	0.11	− 1%	0.976
l_FWM	MANOVA: Wilks-Lambda: *p* = 0.086				
		Post hoc Scheffé test **ΔCN**			
	**fN**	**mN**	**mC**	rel. diff.	*p* value
PME	2.22	2.23	2.19	− 2%	0.934
Pi	0.66	0.60	0.56	− 8%	0.666
PDE	2.14	2.27	2.30	1%	0.958
PCr	3.33	3.73	3.47	− 7%	0.112
ATP	2.35	2.30	2.08	− 10%	0.158
pH	7.00	7.00	7.00	− 0.1%	0.839
Mg	0.11	0.11	0.11	− 2%	0.909

Absolute mean concentration values (in mmol/kg) and pH in left and right-sided voxels for fN, mN, and mC. Left half and right half of the FGM voxel were not evaluated separately

ΔCN indicates the relative difference of metabolite values between mN and mC: $\Delta \mathrm{CN}=\frac{\left(\mathrm{mC}-\mathrm{mN}\right)}{\mathrm{mN}}$

The Wilks-Lambda test reflects the overall effect of the three groups on all seven metabolite values included in the MANOVA. NAD was excluded because of too many low-quality data. Post hoc Scheffé test was used for paired comparison of groups

*p* values < 0.05 are marked in bold. *Values that remained significant after multiple comparisons correction

**Table 2:** Results of 31P MRS, comparison between hemispheres

TH	RM MANOVA: *p* = 0.000*					
	Paired comparison between hemispheres:					
	**fN**		**mN**		**mC**	
	Δrl: *p* = 0.255		Δrl: *p* = 0.000*		Δrl: *p* = 0.008*	
	rel. diff.	*p* value (*t*-test)	rel. diff.	*p* value (*t*-test)	rel. diff.	*p* value (*t*-test)
PME	− 1%	0.844	− 7%	0.013*	− 2%	0.382
Pi	− 5%	0.213	− 5%	0.168	− 7%	0.012*
PDE	− 1%	0.771	− 1%	0.567	− 2%	0.189
PCr	− 1%	0.373	− 8%	0.000*	− 3%	0.002*
ATP	0%	0.785	− 2%	0.178	− 4%	**0.045**
pH	0.0%	0.722	0.0%	0.838	0.0%	0.782
Mg	− 5%	0.081	0%	0.891	1%	0.603
BG	RM MANOVA: *p* = 0.000*					
	Paired comparison between hemispheres:					
	**fN**		**mN**		**mC**	
	Δrl: *p* = 0.153		Δrl: *p* = **0.030**		Δrl: *p* = 0.116	
	rel. diff.	*p* value (*t*-test)	rel. diff.	*p* value (*t*-test)	rel. diff.	*p* value (*t*-test)
PME	− 10%	**0.049**	− 6%	0.134	− 8%	**0.013**
Pi	− 15%	0.063	− 10%	0.212	− 16%	0.004*
PDE	10%	**0.017**	6%	0.304	7%	**0.037**
PCr	− 5%	**0.017**	− 17%	0.000*	− 6%	**0.008**
ATP	− 2%	0.534	2%	0.441	− 4%	**0.020**
pH	− 0.2%	0.224	− 0.1%	0.234	0.0%	0.739
Mg	0%	0.956	− 2%	0.680	− 2%	0.391
TL	RM MANOVA: *p* = **0.163**					
	Paired comparison between hemispheres:					
	**fN**		**mN**		**mC**	
	Δrl: *p* = 0.917		Δrl: *p* = 0.192		Δrl: *p* = 0.238	
	rel. diff.	*p* value (*t*-test)	rel. diff.	*p* value (*t*-test)	rel. diff.	*p* value (*t*-test)
PME	− 4%	0.422	2%	0.677	1%	0.673
Pi	− 5%	0.627	− 12%	0.156	− 10%	0.080
PDE	7%	0.177	19%	0.007*	18%	0.000*
PCr	− 3%	0.331	− 3%	0.409	0%	0.920
ATP	1%	0.647	4%	0.300	− 1%	0.481
pH	0.1%	0.456	0.0%	0.906	− 0.1%	0.272
Mg	9%	0.093	3%	0.646	1%	0.890
FWM	RM MANOVA: *p* = 0.011*					
	Paired comparison between hemispheres:					
	**fN**		**mN**		**mC**	
	Δrl: *p* = 0.149		Δrl: *p* = 0.058		Δrl: *p* = 0.220	
	rel. diff.	*p* value (*t*-test)	rel. diff.	*p* value (*t*-test)	rel. diff.	*p* value (*t*-test)
PME	2%	0.540	5%	0.462	5%	0.172
Pi	− 7%	0.628	5%	0.656	− 3%	0.739
PDE	20%	0.006*	19%	0.001*	6%	0.223
PCr	2%	0.655	− 7%	0.007*	− 2%	0.123
ATP	11%	**0.023**	7%	**0.030**	9%	0.008*
pH	− 0.2%	0.581	− 0.2%	0.156	− 0.2%	0.054
Mg	− 3%	0.428	− 3%	0.380	− 1%	0.903

Difference of mean concentration values and pH between right and left hemisphere for fN, mN, and mC. Relative differences Δrl were calculated as $\Delta \mathrm{rl}=\frac{\left(\mathrm{right}-\mathrm{left}\right)}{\mathrm{left}}$. For the FGM voxel, no side difference could be determined because the left half and right half of the voxel were not evaluated separately. The RM MANOVA reflects the overall hemispheric effect of the three groups on all seven metabolite values included in the MANOVA. NAD was excluded because of too many low-quality data. Paired RM MANOVA was used to investigate hemispheric effects for each group separately. Hemispheric differences for single metabolites were evaluated by Student’s paired *t*-test. *p* values < 0.05 are marked in bold. *Values that remained significant after multiple comparison correction

**Table 3:** Results of 1H MRS, comparison between groups

FGM	MANOVA: Wilks-Lambda: *p* = **0.047**				
		Post hoc Scheffé test **ΔCN**			
	**fN**	**mN**	**mC**	rel. diff.	*p* value
tNAA	22.6	22.8	22.1	− 3%	0.759
tCr	15.5	16.0	16.2	2%	0.984
tCho	4.1	4.5	4.6	1%	0.973
r_TL	MANOVA: Wilks-Lambda: *p* = 0.480				
		Post hoc Scheffé test **ΔCN**			
	**fN**	**mN**	**mC**	rel. diff.	*p* value
tNAA	16.1	15.9	15.2	− 4%	0.846
tCr	10.3	10.3	10.2	− 2%	0.950
tCho	3.0	3.3	3.1	− 5%	0.978
r_TH	MANOVA: Wilks-Lambda: *p* = 0.455				
		Post hoc Scheffé test **ΔCN**			
	**fN**	**mN**	**mC**	rel. diff.	*p* value
tNAA	14.0	14.8	13.4	− 10%	0.500
tCr	8.5	9.2	7.9	− 14%	0.712
tCho	2.4	2.7	2.5	− 6%	0.610
r_FWM	MANOVA: Wilks-Lambda: *p* = 0.593				
		Post hoc Scheffé test **ΔCN**			
	**fN**	**mN**	**mC**	rel. diff.	*p* value
tNAA	14.7	15.7	14.7	− 6%	0.272
tCr	8.2	8.8	8.6	− 2%	0.997
tCho	2.7	2.9	2.8	− 2%	0.962

Absolute mean concentration values (in mmol/kg) for fN, mN, and mC

ΔCN indicates the relative group difference of metabolite values between male non-consumers (mN) and male cannabis-consumers (mC):$\Delta \mathrm{CN}=\frac{\left(\mathrm{mC}-\mathrm{mN}\right)}{\mathrm{mN}}$

The Wilks-Lambda test reflects the overall effect of the three groups on all three metabolites included in the MANOVA. Post hoc Scheffé test was used for paired comparison of groups. *p* values < 0.05 are marked in bold

### Phosphorus MRS results

Statistical analysis with MANOVA showed overall significant group differences of metabolic values only in the r_TH (p=0.014) and r_TL (*p* = 0.034) (Table 1). These results were no longer significant after Bonferroni correction. For some individual metabolites, concentration differences were found by post hoc tests comparing mN with fN and mN with mC, respectively.

#### Differences between male non-users (mN) and female non-users (fN): Δmf

ATP levels in male non-users tended to be lower than in females in nearly all regions, most noticeable in the thalamus (Δmf = − 11% in r_TH and − 10% in l_TH) (Fig. 2) where Δmf was negative for all other metabolites, too. MANOVA revealed significant differences for PME (*p* = 0.014) and PCr (*p* = 0.018) in the right thalamus, although this result did not survive Bonferroni correction.

Males tended to have lower PME mainly in the thalamus but higher PDE values in most regions. Pi was lower in the frontal lobe; pH tended to be slightly higher in males, particularly in the right thalamus and the right FWM. The higher PCr value for males (Δmf = + 12%) in the l_FWM (*p* = 0.018) was no longer significant after Bonferroni correction.

#### Differences between male cannabis users (mC) and male non-users (mN): ΔCN

Cannabis users had consistently lower ATP and Pi levels in all regions (Fig. 3). PDE values tended to be lower in mC except for the thalamus. pH levels were slightly reduced in most regions. None of these differences reached statistical significance. In the mC group, PCr was lower in the left BG (ΔCN = − 10 %, *p* = 0.015) and in the left FWM (− 7%), but this result was no longer significant after Bonferroni correction.

#### Hemispheric differences

Statistical analysis with RM MANOVA showed overall hemispheric asymmetries of metabolic values in TH (*p* = 0.000), BG (*p* = 0.000), and the FWM (*p* = 0.011) (Table 2) remaining significant even after Bonferroni correction. Comparing hemispheres in each group separately, significant overall side effects were found only in the thalamus for mN (*p* = 0.000) and mC (*p* = 0.008), where Δrl was negative for all metabolites (Fig. 4).

Looking at single metabolites, PCr and Pi values appeared overall lower on the right side compared to the left. ATP concentration was significantly higher in the right FWM than in the left. PME tended to be lower on the right side in TH and BG, whereas PDE was higher in the right hemisphere except for the thalamus.

In total, hemispheric differences of comparable extent could be detected in all three groups. For PCr, however, the side effect was strikingly larger in the mN group than in fN and mC, most notably in the basal ganglia (Δrl = − 17% for mN).

### Proton MRS results

Statistical analysis with MANOVA revealed overall significant group differences of proton metabolic values only in FGM (*p* = 0.047) (Table 3), but this result remained no longer significant after Bonferroni correction.

#### Differences between male non-users (mN) and female non-users (fN): Δmf

Among non-users, males tended to have overall higher levels of proton metabolites than females (Fig. 5a), most pronounced for Cho (Δmf = + 11%) in the FGM.

#### Differences between male cannabis users (mC) and male non-users (mN): ΔCN

Compared to mN, proton metabolite data of mC showed a tendency of overall slightly lower concentrations (Fig. 5b) without reaching significance.

### Overview of the results

In summary, we could not find statistically significant differences of metabolite concentrations in the brain of male cannabis-users compared to male non-users, although the data showed some tendencies.

mC showed a reduction in ATP (− 3 to − 12%) and Pi (− 2 to − 15%) in all evaluated regions. PCr concentrations were reduced only in the left BG, the left TH, and in the left FWM. For proton metabolites (tNAA, tCr, and tCho), mC tended to have slightly lower values than mN.

Some differences of metabolite values could also be detected between male and female non-users. An overall lower ATP concentration (− 2 to − 11%) was observed in mN compared to fN. In the TH region, males had generally lower metabolite concentrations than females. Proton metabolites tended to be higher in males.

Hemispheric comparison revealed statistically significant asymmetries of phosphorus metabolite values between right and left. PCr and Pi had a generally lower level in the right hemisphere, most strikingly in the BG (up to − 17%). We could not find consistent discrepancies of lateralization between the groups except for PCr which exhibited a much larger asymmetry in male non-users than in fN and mC.

## Discussion

A review of the MRS literature concerning cannabis abuse clearly shows the paucity of data in this field [53–58]. To date, few 1H MRS studies characterizing proton neurometabolite concentrations in cannabis users have been published, but 31P MRS data are completely lacking until now. This study represents the first attempt to combine 1H MRS and 31P MRS in order to evaluate and compare neurometabolism in young cannabis users by performing an absolute quantification of several metabolites in different anatomic regions of the brain.

Proton spectroscopy studies dealing with cannabis consumption that have been published so far have focused on regions such as the frontal lobe, basal ganglia, hippocampus, and temporal lobe. Reduced NAA is the most frequently observed finding in cannabis users [59–62]. Particularly in the youngest subjects, reduced NAA levels were detected in frontal lobe regions, including the dorsolateral prefrontal cortex, anterior cingulate gyrus, inferior frontal gyrus, and midfrontal gray matter. Greater amount of cannabis use was associated with lower NAA and lower Cho. These results are confirmed by our study which found slightly (but not significantly) reduced NAA and Cho levels in all examined regions in the mC group.

N-acetylaspartate (NAA) is the second-most-concentrated molecule in the brain after the amino acid glutamate; its physiological function though still remains not absolutely clear [63]. NAA is detectable not only in neurons in the adult brain [64] but also in oligodendrocytes and myelin [65].

As a contributor to energy production from the amino acid glutamate, NAA correlates with the integrity of neuronal mitochondrial function [66]. Reduction of NAA concentration in the brain of cannabis users as observed both in previous and in our study might reflect neurotoxic effects of cannabis compromising neural viability.

Choline has many functions within humans and other organisms with the key feature of serving as a synthetic precursor for phospholipids that form cell membranes, the neurotransmitter acetylcholine, and trimethylglycine. Lower Cho refers to a reduced membrane turnover or increased cellular/neuronal senescence. Subsequently, lowered acetylcholine concentrations interfere with neuronal integrity, metabolism, cognition, consciousness [67] and are a predisposing factor in neurodegenerative illnesses, e.g., Alzheimer’s disease [68–70].

In our study, we did not find a significant impact of sex on proton metabolite concentrations, but the data showed some tendencies in several regions of the brain. Males (mN) tended to have higher levels than females (fN), especially for creatine and choline. These results could be interpreted in the context of well-known sex differences in brain function and structure [71]. In addition, metabolic effects of menstrual cycle have to be considered [72, 73].

The most interesting result of our 31P MRS measurements is the consistent, but not statistically significant, trend to a reduction of ATP and Pi levels in mC compared to mN. PDE values were decreased mainly in the frontal and in the temporal lobe. Lowering of PCr was observed in the left part of BG, TL, and FWM. ATP, which is provided by oxidative chain reactions on the inner mitochondrial membrane, is essential for the cellular energy supply, especially for brain neurons. PCr serves as a cellular energy reservoir which can quickly provide ATP through hydrolysis. Depletion of ATP and Pi was observed in mC compared to mN which could probably point to an energy shortage in neurons, axons, and the neuroglial cells. As PDE mainly represents phospholipid breakdown products [74], reduced PDE levels as found in the frontal and in the temporal lobe could indicate lower membrane turnover, probably as a result of disturbed phospholipid generation rather than accelerated phospholipid degradation [75, 76]. Cannabis-induced metabolic changes in the TL are of particular interest regarding auditory perception and language processing. The temporal lobe includes many important functions, such as the primary auditory cortex and Wernicke area which represent an integrated part of the speech recognition and speech production; the concrete function is still seen controversial [77]. The reduction of metabolite concentrations (except PME) observed in the TL is consistent with the fMRI study of Winton-Brown et al. 2011 [78] which found an attenuation of temporal auditory activation after administration of THC. Whereas their study showed an increase in psychotic symptoms associated with the attenuation of temporal activation, there were no signs of psychosis in our subject group.

Furthermore, a slight tendency of reduced pH values could be determined in the FGM, FWM, TL, and BG similar to decreased pH values reported in the frontal lobe of patients with bipolar disorder [79]. Reduction of pH could be the result of the above-mentioned energy shortage that leads to an increased anaerobic glycolysis with elevated lactate levels and reduced pH value. As we did not detect lactate in our proton MRS measurements, these presumably increased lactate levels are still very low.

In summary, our 31P MRS results can be interpreted—with all due caution—as an indication of reduced energy supply and decreased membrane turnover particularly in the frontal lobe and in the BG of cannabis users. As shown by several studies, the frontal lobe is an important part of the neuronal network responsible for social function [80], cognitive skills [81, 82], and general intelligence [83]. The BG are associated with the control of movements but also a variety of cognitive and affective functions [84–86]. In conclusion, our findings might help to understand the negative impact of cannabis use on a variety of brain functions observed in long-term cannabis users. In general, the obtained 31P MRS results correlate with the results of the FDG PET findings [28] which showed a decreased glucose uptake in several brain regions of young cannabis users.

Structural T2-weighted images did not reveal any visible correlate to the metabolic changes we found in cannabis users, but several structural brain changes on cellular levels have been found in other studies, for example, the impact of cannabis use on white matter integrity [87], corpus callosum [88], gray matter density [89], and brain tissue composition [16]. Microscopy studies analyzing brain tissue of cannabis users are not available up to now. Thus, more MRI studies are needed to determine whether brain lesions might occur in the long term.

The analysis of sex influence on phosphorus metabolites yielded an inhomogeneous pattern. Major effects were found in the thalamus where mN exhibited overall lower concentrations than fN. ATP values were lower in males than in females in all examined regions. As explained above in the case of proton metabolites, sex-related differences may be partially related to hormonal conditions.

In all of our three subject groups, we found significant asymmetries of phosphorus metabolite concentrations between right and left hemisphere. In general, concentrations of PCr, Pi, and PME are higher on the left side, while PDE levels are lower, indicating an intensified energy metabolism and an elevated rate of membrane synthesis in the left hemisphere compared to the right. These side differences can be explained in the framework of functional and structural lateralization of the brain. As only right-handers were included in the study, we can assume that their left hemisphere is dominant. Complex functions like the control of behavioral structures, movement, language, and cognition are primarily located in the dominant hemisphere [90–92] potentially resulting in an asymmetric distribution of energy and membrane metabolism between the hemispheres, in accordance with our findings.

It is worth noting that the extent of metabolic asymmetry is about the same in all three analyzed subject groups except for PCr. Side differences of PCr concentration are fairly low in fN as well as in mC. In mN, however, the level of PCr was detected to be much lower on the right side than on the (dominant) left, especially in the BG. Behavioral studies have shown reduced left-hemispheric language dominance in schizophrenia as well as in healthy schizotypal subjects [93]. As cannabis use is considered as a risk factor for the development of psychosis, it may also influence the extent of lateralization for specific metabolites. Thus, the reduced asymmetry of PCr values in mC compared to mN might be interpreted in this context.

In conclusion, combined ^1^H/^31^P-MRS showed a trend towards decreased concentrations of Pi, ATP, and PCr in the frontal lobe region, as well as the right and left basal ganglia in young cannabis users compared to non-cannabis users. The results suggest that functional brain disorders observed in long-term cannabis users might be caused by an impairment of the energy metabolism of the brain, interfering neuronal integrity and viability, cognition, motoric, and sensual perception. Some of the results indicate that this impact on brain metabolism might accelerate neuronal senescence and subsequently could be a predisposing factor for neurodegenerative diseases. The extent of the observed metabolite concentration differences between the groups did not reach the level of significance; only the hemispheric asymmetric effects were statistically significant. Thus, more ^31^P-MRS long-term studies are required for verification.

### Limitations

The results presented should be interpreted with caution. Due to imperfect magnetic field homogeneity and relatively small size of ROIs, the SNR of spectroscopic signals is fairly low in some regions of the brain, particularly for those metabolites that always produce relatively small peaks such as PME, PDE, and Pi. Moreover, there was a lack of homogeneity of the mC subject group caused by a wide range of cannabis use. This might be one of the reasons that metabolic differences between cannabis users and non-users did not reach the level of significance. In contrast, metabolic differences between the right and left hemisphere of the brain could be established in all three groups with high significance, because paired comparison within groups is less susceptible to inter-subject variations.

Another limitation concerning our 1H MRS data is caused by the use of a long echo time TE, as we originally focused on the main metabolites NAA, Cho, and Cr. Choosing a value of TE = 135 ms impeded the detection of other relevant metabolites such as glutamine and glutamate which seem to be important in cannabis use, according to recent publication [55].

One additional factor that needs to be considered in our study is the possibly confounding role of smoking. Nicotine consumption (package years) was not balanced between the groups but strongly associated with cannabis use (supplementary_Demographics: suppl_table 1). So, we cannot exclude that nicotine consumption could have contributed to the difference of metabolite values between mN and mC found in this study. On the other hand, to our knowledge, there is no comparable 31P MRS study investigating the influence of nicotine on brain metabolism in young adults but only 1H MRS studies focusing mainly on the anterior cingulate cortex with reported inconsistencies in the findings [94]. Furthermore, the half-life of nicotine in the brain is approximately 1 to 2 h [95–97] temporally restricting the effect on cerebral blood flow and metabolism. In contrast, THC is expected to have a long-term impact on brain metabolism due to its very long half-life of 5–13 days [98]. Moreover, nicotine consumption in the mC group was fairly low with a median of 1.2 py (supplementary_Demographics: suppl_table 1). In order to reduce the potential nicotine effect, subjects were instructed to abstain from smoking on examination day; noncompliance led to exclusion from the study.

As well as moving to enhanced techniques of investigation there is also the need for standardization of the populations being studied and better metabolite quantification. Future studies using 1H MRS should definitely be based on short echo times in order to extend the range of detectable metabolites. Finally, the constantly growing use of cannabis and worries about school performance in young users demand further research in this field.

### Supplementary information

ESM 1 (PDF 768 KB)

ESM 2 (PDF 2.20 MB)

ESM 3 (PDF 1.73 MB)

ESM 4 (PDF 2.11 MB)

ESM 5 (PDF 1.33 MB)

## Data Availability

The data that support the findings of this study are available from the corresponding author upon reasonable request.

## Abbreviations

∆9-THC: Delta-9-tetrahydrocannabinol
1H: Proton
31P: Phosphorus
3T: 3 tesla
ADHD: Attention-deficit hyperactivity disorder
ADP: Adenosine diphosphate
AMARES: Advanced method for accurate, robust, and efficient spectral fitting
AMP: Adenosine monophosphate
BDI: Beck’s Depression Inventory
BG: Basal ganglia
CB 1: Cannabinoid receptor 1
CBD: Cannabidiol
Ch: Cytosolic choline
Cr: Creatine
CSI: Chemical-shift-imaging
CUDIT: Cannabis use disorder identification test
fC: Female cannabis users
FGM: Frontal gray matter
FID: Free-induction-decay
fN: Female cannabis non-users
FWM: Frontal white matter
M: Gray matter
GPC: Glycerol-phosphocholine
GPE: glycerol-phosphoethanolamine
jMRUI: Java-based graphical user interface for the magnetic resonance user interface (MRUI)
l_BG: Left basal ganglia
l_FWM: Left frontal WM
l_TH: Left thalamus
l_TL: Left temporal lobe
mC: Male cannabis users
mN: Male cannabis non-users
MPRAGE: Magnetization prepared-rapid gradient echo
MRS: Magnetic resonance spectroscopy
NAA: N-acetyl-aspartate
NAD: Nicotinamide adenine dinucleotide
NC: Cannabis non-users
PCr: Phosphocreatine
PDE: Phosphodiester
PE: Phosphatidylethanolamine
Pi: Inorganic phosphate
PME: Phosphomonoesters
ppm: Parts per million
r_BG: Right basal ganglia
r_FWM: Right frontal WM
r_TH: Right thalamus
r_TL: Right temporal lobe
ROI: Region of interest
SD: Standard deviation
tCho: Total choline
tCr: Total creatine
TE: Echo time
TH: Thalamus
TL: Temporal lobe
tNAA: Total n-acetyl-aspartate
TR: Repetition time
VOI: Volume of interest
WM: White matter
