# Supplementary material for: Impact of cannabis use on brain metabolism using 31P and 1H magnetic resonance spectroscopy
Source: Neuroradiology. 2023 Sep 22;65(11):1631–48. doi: 10.1007/s00234-023-03220-y (PMC10567915; doi:10.1007/s00234-023-03220-y)
Supplement: Supplementary file 1 — (PDF 768 KB) [file 234_2023_3220_MOESM1_ESM.pdf]

Impact of cannabis use on brain metabolism using ^{31}P and ^{1}H magnetic resonance spectroscopy

Maximilian Fenzl[1] (ORCID 0000-0003-1011-2754) · Martin Backens[1] (ORCID 0000-0002-3414-696X) · Silviu Bodea[2] · Miriam Wittemann[3] · Florian Werler[4] · Jule Brielmaier[5] · Robert Christian Wolf[4] (ORCID 0000-0002-5358-5212) ·Wolfgang Reith[1]

1. Institute of Neuroradiology, Saarland University, 66421 Homburg, Germany
2. Helmholtz Zentrum Munich, German Research Center for Environmental Health Institute of Biological and Medical Imaging, 85748 Munich, Germany
3. Department of Psychiatry and Psychotherapy, Saarland University, 66421 Homburg, Germany
4. Department of General Psychiatry at the Center for Psychosocial Medicine, Heidelberg University, 69115 Heidelberg, Germany
5. Department of Obstetrics and Gynecology, RKH Clinic Ludwigsburg, 71640 Ludwigsburg, Germany

Suppl_Table1: Demographics

**Demographics and clinical scores**	**fN** percentile			**mN** percentile			**mC** percentile			**p value ΔCN**
	**25**	**median**	**75**	**25**	**median**	**75**	**25**	**median**	**75**	
number of subjects		21			26			40		
Age, years	21.7	22.9	23.8	23.3	24.6	26.5	21.2	23.1	25.7	0.172
Years of education	12.0	13.0	16.0	12.0	12.8	16.5	12.0	13.0	15.8	0.991
Tobacco use, py	0.0	0.0	0.0	0.0	0.0	0.0	0.0	1.2	5.0	**0.001**
BDI	1.0	3.0	5.0	0.0	2.0	4.0	1.5	4.0	8.0	0.143
CUDIT							8.5	12.0	18.5	
Duration of use, years							0.0	2.0	4.5	
Onset use, age							15.8	17.8	20.0	
lifetime joints (x1000)							0.4	1.0	5.5	
Current use, days/week							1.50	4.0	7.00	
Current use, g/week							0.33	2.6	5.25	

Tobacco py: number of packs of cigarettes smoked per day multiplied by the number of years the person has smoked
BDI: Beck depression inventory
CUDIT: Cannabis use identification test
p-values resulting from Mann-Whitney U test between mN and mC groups (ΔCN). p-values < 0.05 are marked in bold.

Demographics and clinical assessment scores of study subjects
