# Supplementary material for: Impact of cannabis use on brain metabolism using 31P and 1H magnetic resonance spectroscopy
Source: Neuroradiology. 2023 Sep 22;65(11):1631–48. doi: 10.1007/s00234-023-03220-y (PMC10567915; doi:10.1007/s00234-023-03220-y)
Supplement: Supplementary file 2 — (PDF 2.20 MB) [file 234_2023_3220_MOESM2_ESM.pdf]

Impact of cannabis use on brain metabolism using ^{31}P and ^{1}H magnetic resonance spectroscopy

Maximilian Fenzl[1] (ORCID 0000-0003-1011-2754) · Martin Backens[1] (ORCID 0000-0002-3414-696X) · Silviu Bodea[2] · Miriam Wittemann[3] · Florian Werler[4] · Jule Brielmaier[5] · Robert Christian Wolf[4] (ORCID 0000-0002-5358-5212) ·Wolfgang Reith[1]

1. Institute of Neuroradiology, Saarland University, 66421 Homburg, Germany
2. Helmholtz Zentrum Munich, German Research Center for Environmental Health Institute of Biological and Medical Imaging, 85748 Munich, Germany
3. Department of Psychiatry and Psychotherapy, Saarland University, 66421 Homburg, Germany
4. Department of General Psychiatry at the Center for Psychosocial Medicine, Heidelberg University, 69115 Heidelberg, Germany
5. Department of Obstetrics and Gynecology, RKH Clinic Ludwigsburg, 71640 Ludwigsburg, Germany

Suppl_Fig1: Voxel placement for Phosphorus MRS

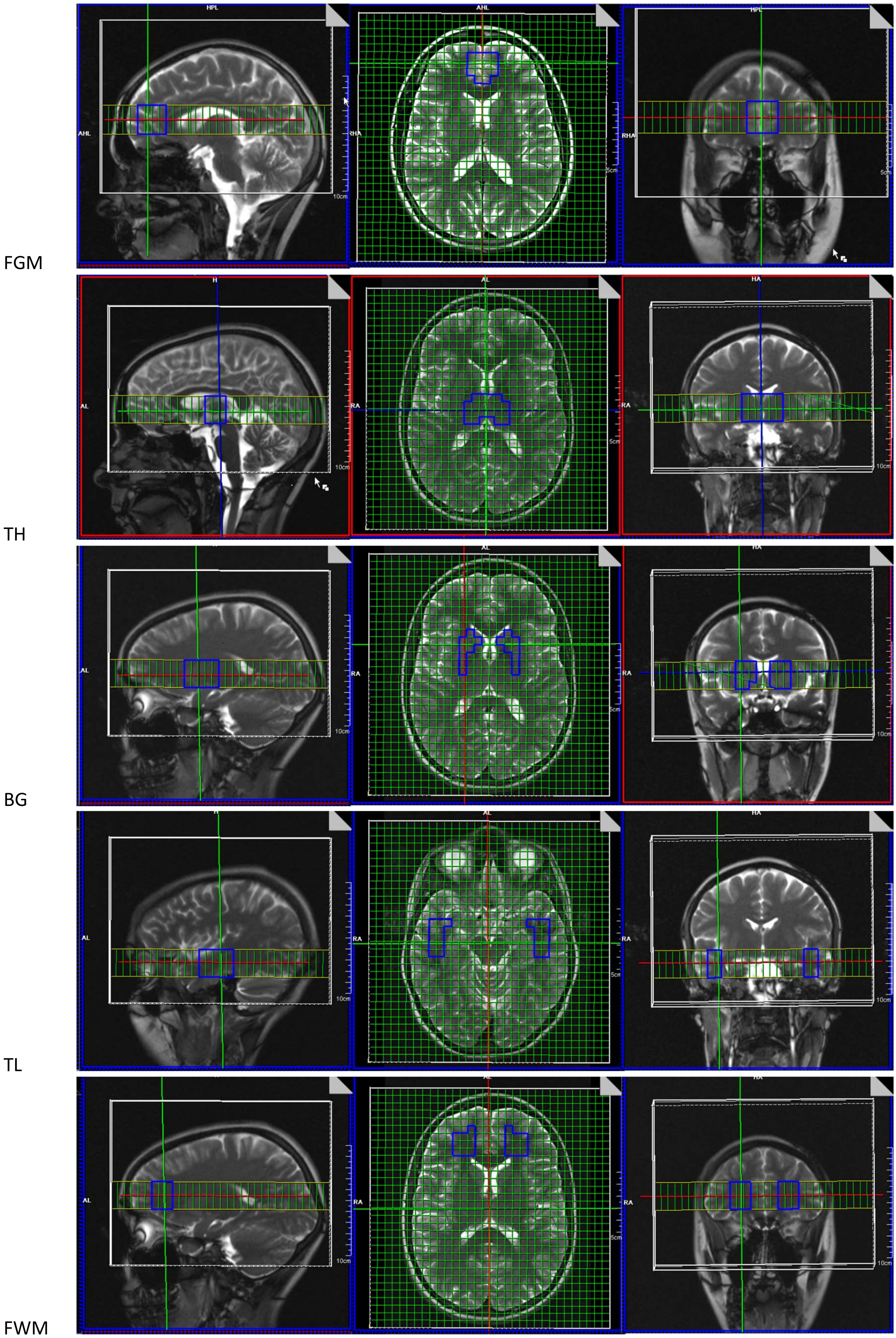

Graphical examples of voxel placement for Phosphorus MRS for each VOI of the right and left hemisphere

Suppl_Fig2:   Evaluation of Phosphorus Spectra – examples

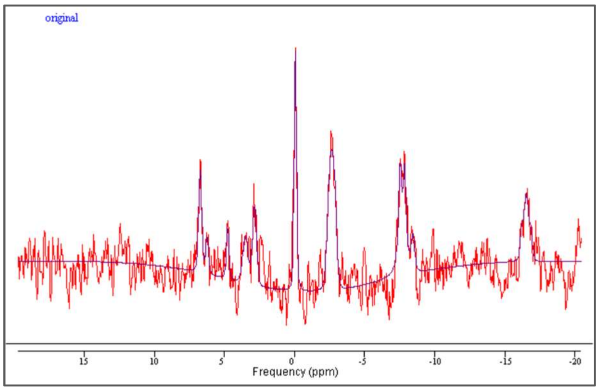

FGM

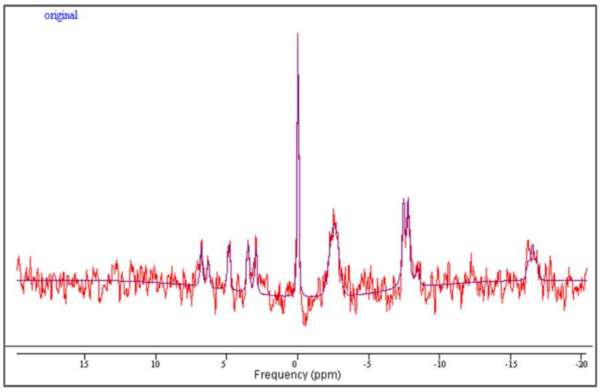

r_TH

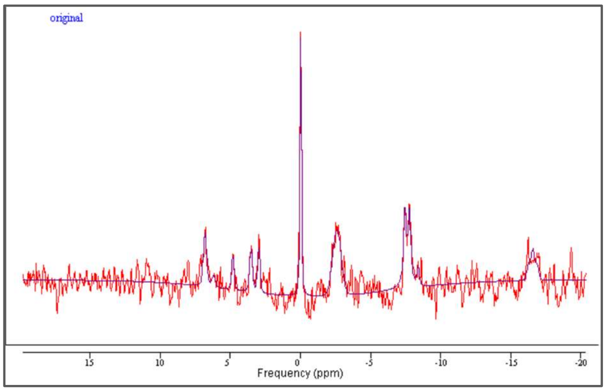

l_TH

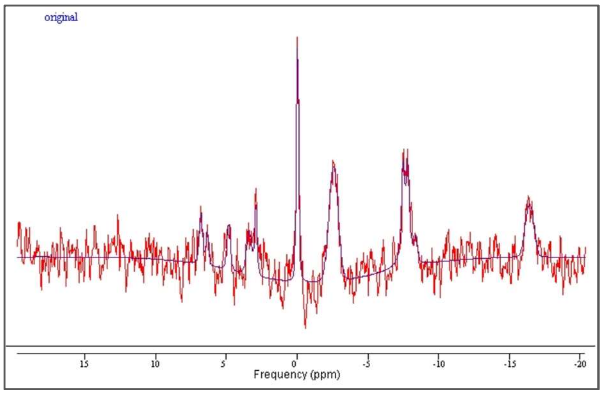

r_BG

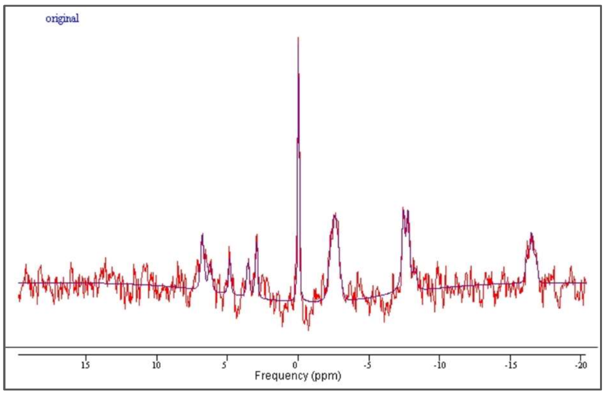

l_BG

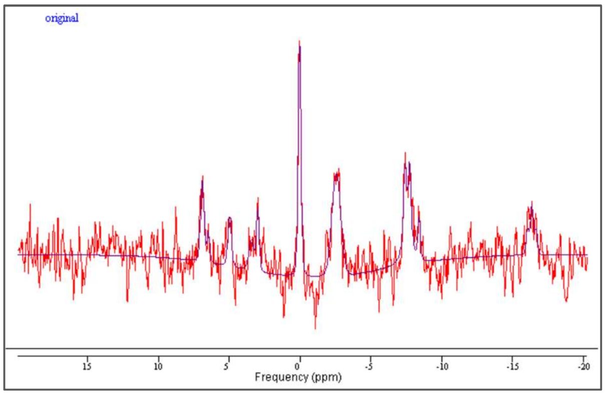

r_TL

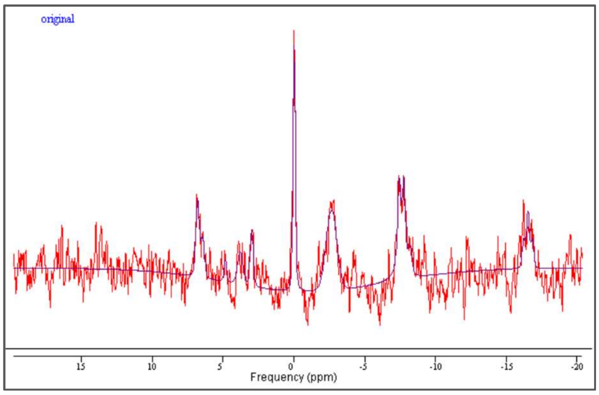

l_TL

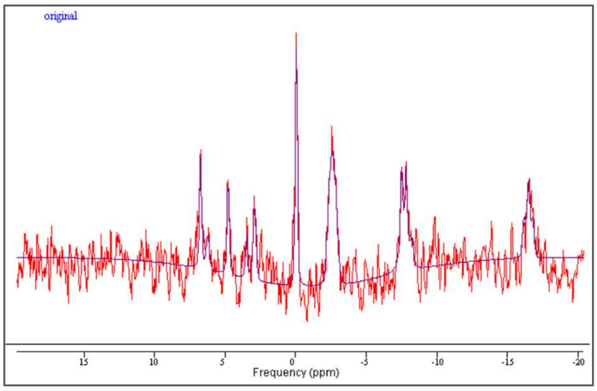

r_FWM

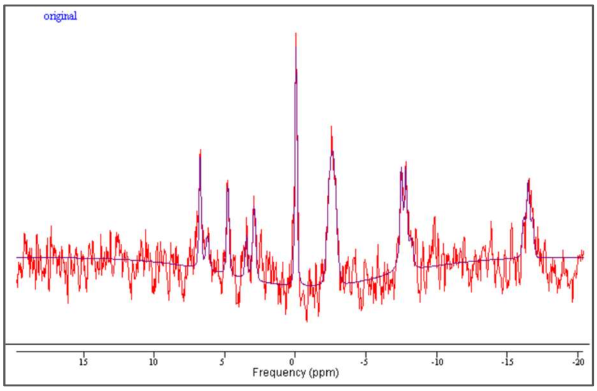

l_FWM

Representative phosphorus spectra from each VOI of the right and left hemisphere with metabolite fit using AMARES (jmrui software)

Suppl_Table2: Phosphorus MRS Data Quality – spreadsheet

mean ± SD	voxel size (ml) - right		
	fN	mN	mC
FGM	15.9 ± 2.4	15.8 ± 2.3	15.8 ± 2.2
r_TH	10.7 ± 0.8	10.9 ± 0.7	10.7 ± 0.9
r_BG	10.7 ± 0.8	10.4 ± 0.6	10.7 ± 0.8
r_TL	12.1 ± 0.3	12.2 ± 0.7	12.3 ± 0.6
r_FWM	9.6 ± 1.1	9.6 ± 1.0	9.5 ± 1.0

mean ± SD	voxel size (ml) - left		
	fN	mN	mC
FGM	15.9 ± 2.4	15.8 ± 2.3	15.8 ± 2.2
l_TH	10.7 ± 0.7	11.0 ± 0.7	10.9 ± 0.7
l_BG	10.6 ± 0.9	10.3 ± 0.7	10.7 ± 0.9
l_TL	12.0 ± 0.3	12.1 ± 0.6	12.3 ± 0.6
l_FWM	9.5 ± 1.1	9.5 ± 0.9	9.5 ± 0.9

mean ± SD	SNR - right		
	fN	mN	mC
FGM	5.1 ± 2.3	5.0 ± 1.8	4.5 ± 1.6
r_TH	11.8 ± 4.4	13.3 ± 3.3	12.1 ± 4.2
r_BG	8.3 ± 3.0	9.2 ± 2.8	8.4 ± 2.9
r_TL	6.9 ± 1.7	8.0 ± 2.2	7.3 ± 2.1
r_FWM	4.9 ± 1.7	5.9 ± 1.6	4.8 ± 1.2

mean ± SD	SNR - left		
	fN	mN	mC
FGM	5.1 ± 2.3	5.0 ± 1.8	4.5 ± 1.6
l_TH	12.4 ± 4.0	13.6 ± 2.7	12.7 ± 4.2
l_BG	9.8 ± 3.8	9.9 ± 2.6	9.3 ± 3.2
l_TL	7.2 ± 1.9	8.4 ± 2.3	7.5 ± 2.2
l_FWM	4.9 ± 1.4	5.9 ± 1.8	5.0 ± 1.3

mean ± SD	PCr line width - right		
	fN	mN	mC
FGM	14.6 ± 4.1	14.4 ± 4.1	14.3 ± 3.6
r_TH	8.3 ± 2.6	6.9 ± 1.4	7.5 ± 1.9
r_BG	11.0 ± 2.5	9.6 ± 2.5	10.4 ± 3.0
r_TL	8.6 ± 1.7	8.6 ± 1.9	8.2 ± 1.6
r_FWM	15.7 ± 3.3	13.4 ± 3.5	15.4 ± 2.9

mean ± SD	PCr line width - left		
	fN	mN	mC
FGM	14.6 ± 4.1	14.4 ± 4.1	14.3 ± 3.6
l_TH	8.2 ± 2.2	7.2 ± 1.5	7.5 ± 1.7
l_BG	10.9 ± 3.3	10.3 ± 2.0	10.0 ± 2.0
l_TL	12.1 ± 2.7	11.9 ± 2.5	12.9 ± 3.0
l_FWM	16.3 ± 3.3	14.0 ± 3.2	14.4 ± 3.2

Mean values and standard deviation of voxel size, SNR and PCr line width of phosphorus spectra in each VOI of the right and left hemisphere for the three subject groups

Suppl_Fig3: Phosphorus MRS Data Quality – bar plot

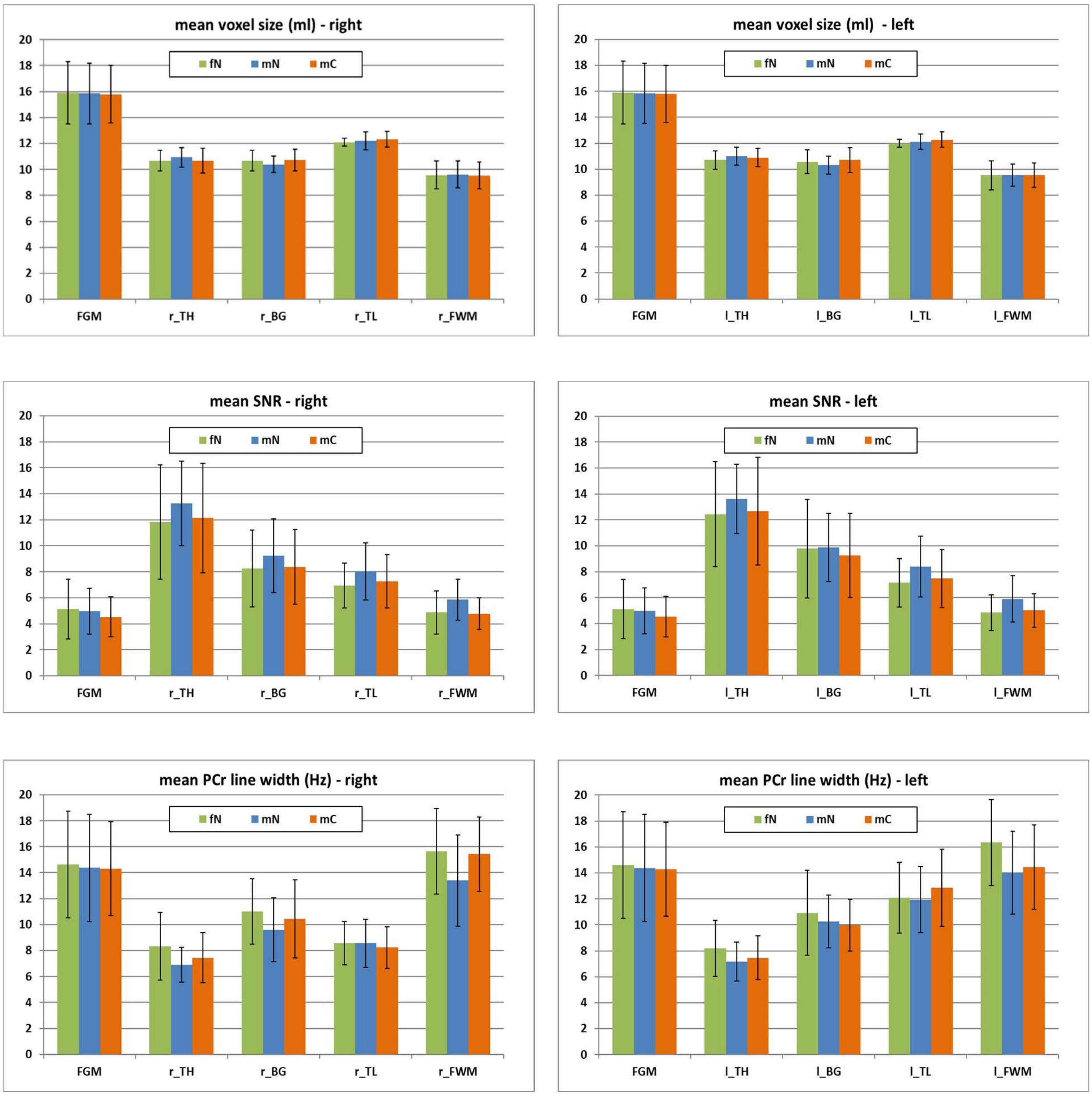

Comparison of mean values of voxel size, SNR and PCr line width of phosphorus spectra in each VOI of the right and left hemisphere for the three subject groups

Suppl_Fig4: Phosphorus MRS Data Quality – box plot

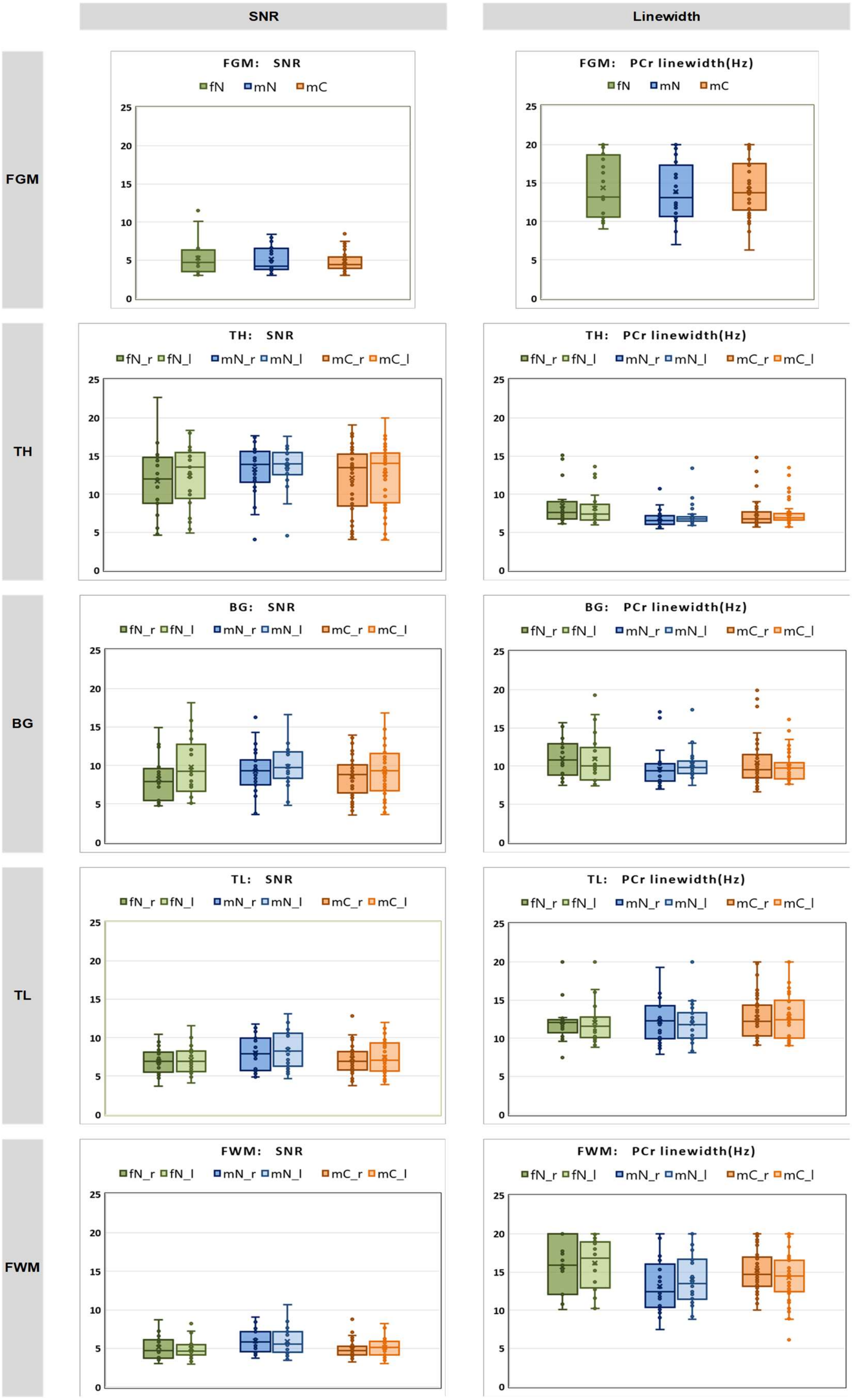

Box plots describing the variation of SNR and PCr line width of phosphorus spectra in each VOI of the right and left hemisphere for the three subject groups
