# Supplementary material for: Impact of cannabis use on brain metabolism using 31P and 1H magnetic resonance spectroscopy
Source: Neuroradiology. 2023 Sep 22;65(11):1631–48. doi: 10.1007/s00234-023-03220-y (PMC10567915; doi:10.1007/s00234-023-03220-y)
Supplement: Supplementary file 3 — (PDF 1.73 MB) [file 234_2023_3220_MOESM3_ESM.pdf]

Impact of cannabis use on brain metabolism using ^{31}P and ^{1}H magnetic resonance spectroscopy

Maximilian Fenzl[1] (ORCID 0000-0003-1011-2754) · Martin Backens[1] (ORCID 0000-0002-3414-696X) · Silviu Bodea[2] · Miriam Wittemann[3] · Florian Werler[4] · Jule Brielmaier[5] · Robert Christian Wolf[4] (ORCID 0000-0002-5358-5212) ·Wolfgang Reith[1]

1. Institute of Neuroradiology, Saarland University, 66421 Homburg, Germany
2. Helmholtz Zentrum Munich, German Research Center for Environmental Health Institute of Biological and Medical Imaging, 85748 Munich, Germany
3. Department of Psychiatry and Psychotherapy, Saarland University, 66421 Homburg, Germany
4. Department of General Psychiatry at the Center for Psychosocial Medicine, Heidelberg University, 69115 Heidelberg, Germany
5. Department of Obstetrics and Gynecology, RKH Clinic Ludwigsburg, 71640 Ludwigsburg, Germany

Suppl_Fig5: Voxel placement for Proton MRS

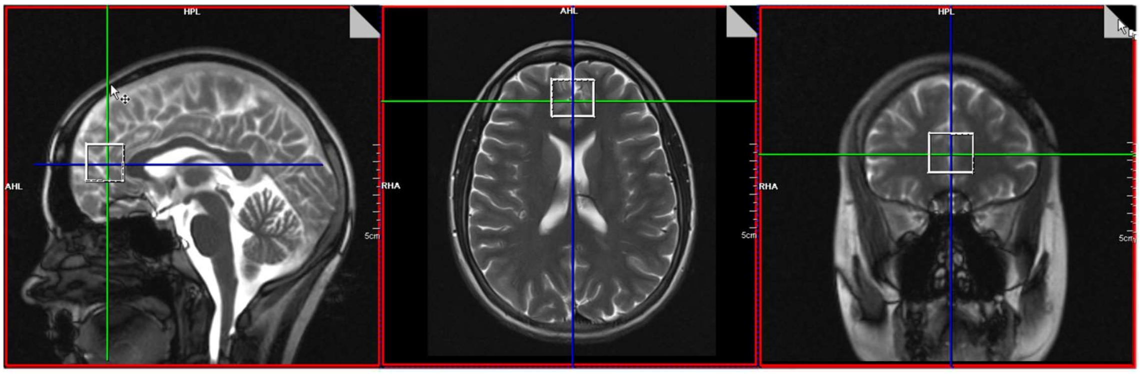

FGM

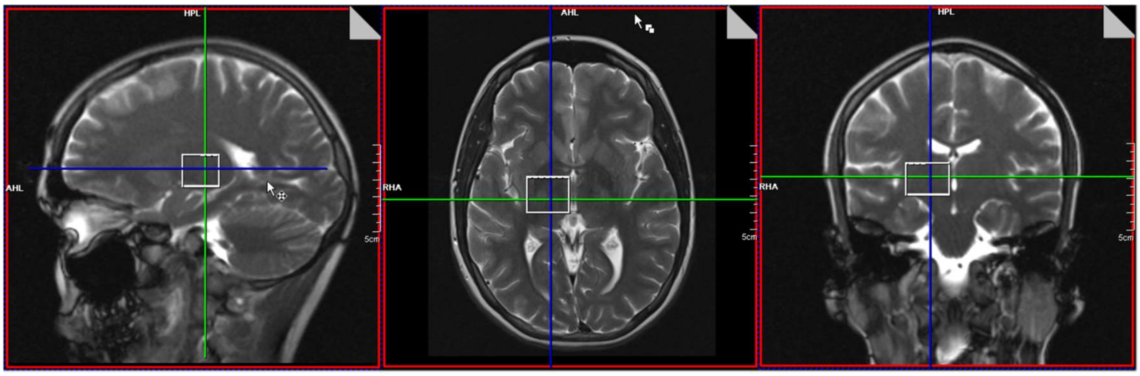

r_TH

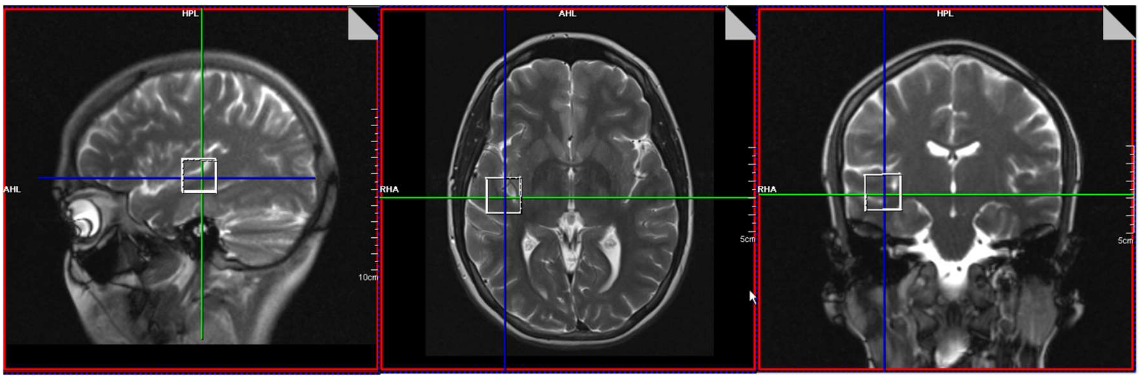

r_BG

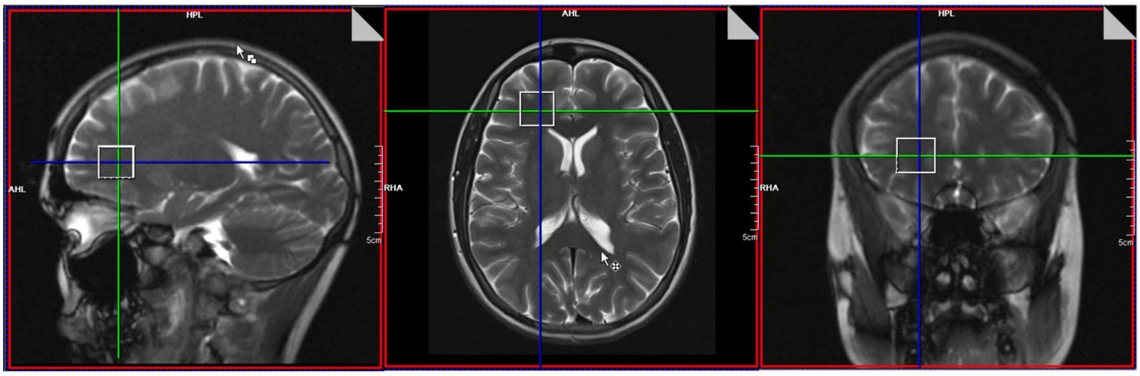

r_FWM

Graphical examples of voxel placement for Proton MRS for each VOI

Suppl_Fig6: Evaluation of Proton Spectra – examples

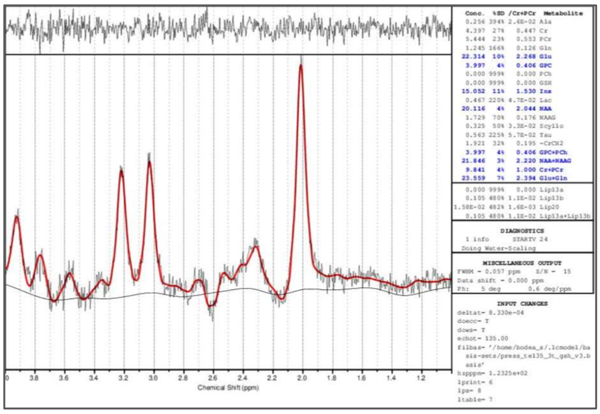

FGM

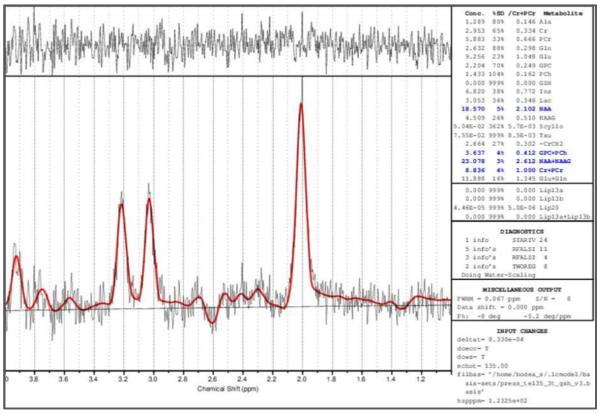

r_TH

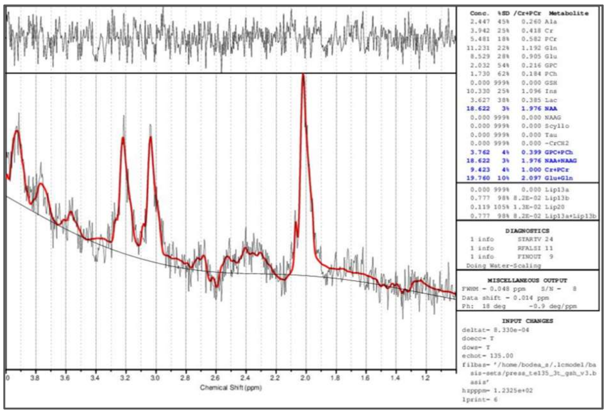

r_TL

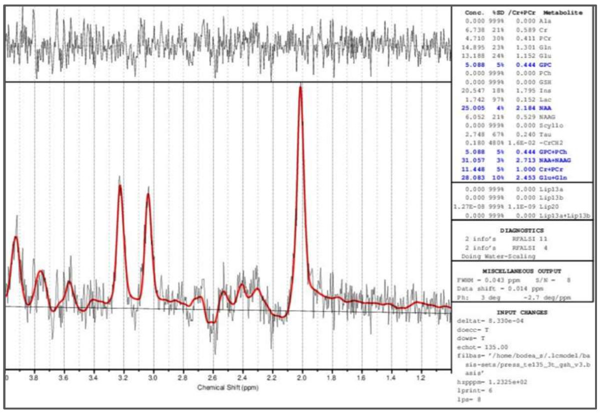

r_FWM

Representative proton spectra from each VOI with metabolite fit using LCModel software

Suppl_Table3: Proton MRS Data Quality - spreadsheet

mean ± SD	voxel size (ml)		
	**fN**	**mN**	**mC**
FGM	14.8 ± 1.4	15.2 ± 0.9	14.5 ± 1.4
r_TH	12.0 ± 1.2	12.0 ± 0.9	11.9 ± 0.9
r_TL	9.9 ± 0.3	10.0 ± 0.1	9.9 ± 0.4
r_FWM	7.6 ± 0.9	7.6 ± 0.6	7.6 ± 0.6

mean ± SD	SNR		
	**fN**	**mN**	**mC**
FGM	11.0 ± 2.5	7.9 ± 3.0	8.3 ± 2.0
r_TH	5.5 ± 1.9	4.7 ± 1.8	4.8 ± 1.3
r_TL	6.8 ± 2.2	5.6 ± 2.2	5.5 ± 1.5
r_FWM	6.2 ± 3.0	5.5 ± 2.6	5.3 ± 1.6

mean ± SD	water line width (Hz)		
	**fN**	**mN**	**mC**
FGM	6.5 ± 1.7	7.2 ± 2.5	6.9 ± 1.7
r_TH	10.0 ± 1.9	10.2 ± 2.1	9.7 ± 1.7
r_TL	7.2 ± 2.4	7.5 ± 1.7	7.9 ± 1.8
r_FWM	7.7 ± 2.1	7.8 ± 1.9	8.0 ± 2.1

Mean values and standard deviation of voxel size, SNR and water line width of proton spectra in each VOI for the three subject groups

Suppl_Fig7: Proton MRS Data Quality – bar plot

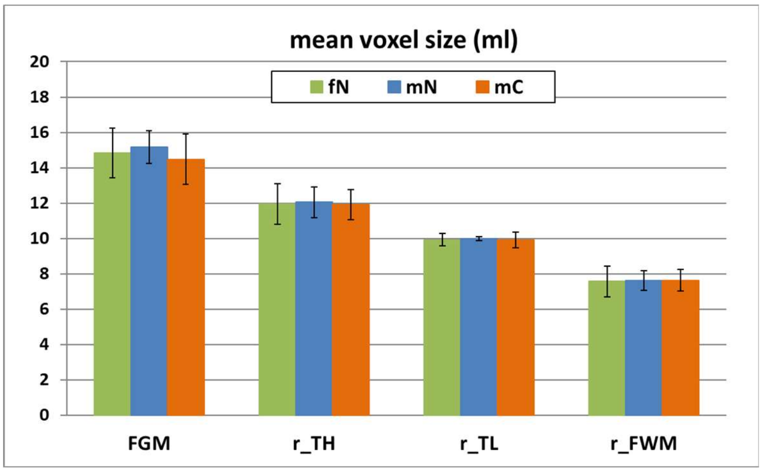

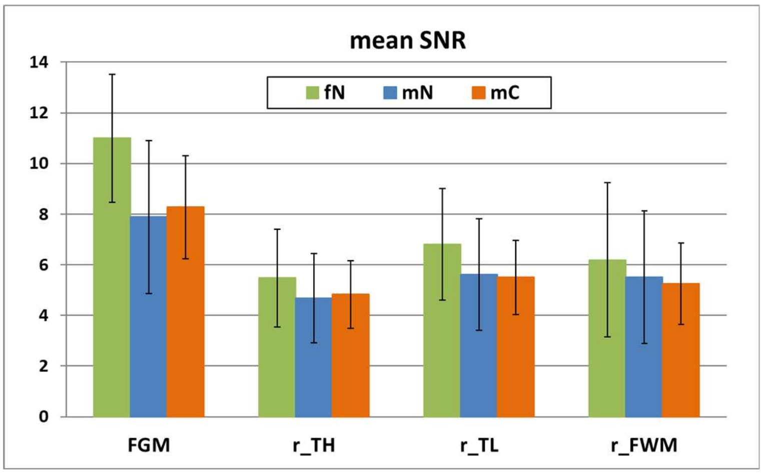

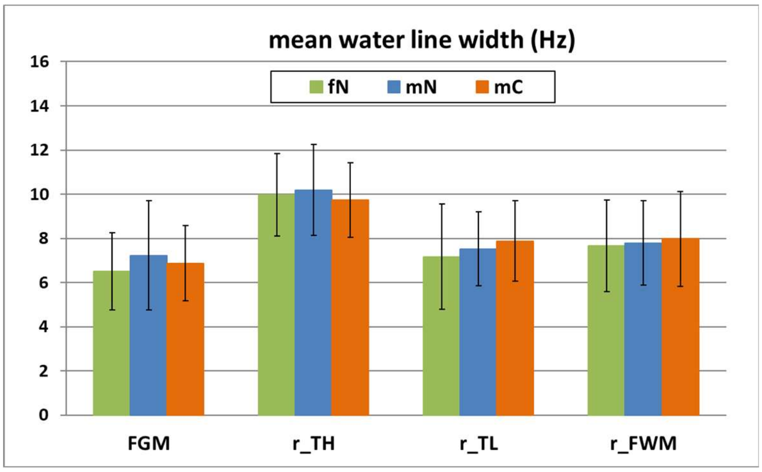

Comparison of mean values of voxel size, SNR and water line width of proton spectra in each VOI for the three subject groups

Suppl_Fig8: Proton MRS Data Quality – box plot

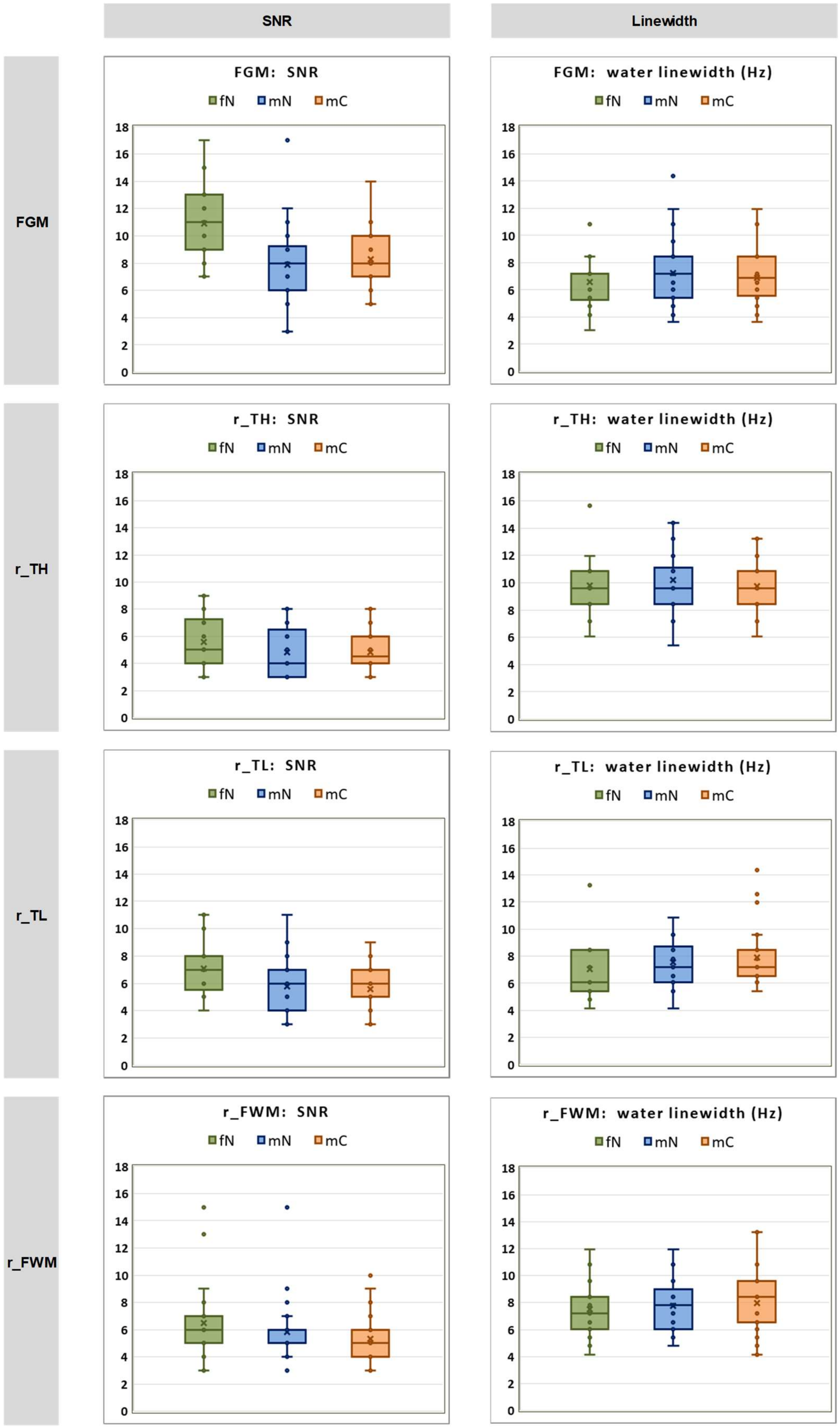

Box plots for SNR and water line width of proton spectra in each VOI for the three subject groups
