# Supplementary material for: Impact of cannabis use on brain metabolism using 31P and 1H magnetic resonance spectroscopy
Source: Neuroradiology. 2023 Sep 22;65(11):1631–48. doi: 10.1007/s00234-023-03220-y (PMC10567915; doi:10.1007/s00234-023-03220-y)
Supplement: Supplementary file 4 — (PDF 2.11 MB) [file 234_2023_3220_MOESM4_ESM.pdf]

Impact of cannabis use on brain metabolism using ^{31}P and ^{1}H magnetic resonance spectroscopy

Maximilian Fenzl[1] (ORCID 0000-0003-1011-2754) · Martin Backens[1] (ORCID 0000-0002-3414-696X) · Silviu Bodea[2] · Miriam Wittemann[3] · Florian Werler[4] · Jule Brielmaier[5] · Robert Christian Wolf[4] (ORCID 0000-0002-5358-5212) ·Wolfgang Reith[1]

1. Institute of Neuroradiology, Saarland University, 66421 Homburg, Germany
2. Helmholtz Zentrum Munich, German Research Center for Environmental Health Institute of Biological and Medical Imaging, 85748 Munich, Germany
3. Department of Psychiatry and Psychotherapy, Saarland University, 66421 Homburg, Germany
4. Department of General Psychiatry at the Center for Psychosocial Medicine, Heidelberg University, 69115 Heidelberg, Germany
5. Department of Obstetrics and Gynecology, RKH Clinic Ludwigsburg, 71640 Ludwigsburg, Germany

Suppl_Table4: T1 relaxation for Phosphorus MRS

	PE	PC	Pi	GPE	GPC	PCr	ATP	NAD	Phantom
**T1 (ms)**	4000	3300	2200	4000	2700	3500	1000	1000	4000
**R_P**	0.36	0.4	0.51	0.36	0.46	0.39	0.71	0.71	0.36

T1 values used for relaxation correction of phosphorus metabolite values and calculated relaxation factors R_P

Suppl_Table5: Results of 31P MRS - Comparison between groups

**FGM**	**overall comparison of groups (multivariate)** Statistics MANOVA: Wilks-Lambda p = 0.175							
		**pairwise group comparison** (post-hoc Scheffé test)						
	omnibus p-value	**fN (n=20)** mean ± SD	**Δmf** rel. diff.	**Δmf** p-value	**mN (n=24)** mean ± SD	**ΔCN** rel. diff.	**ΔCN** p-value	**mC (n=34)** mean ± SD
**PME**	0.764	2,70 ± 0,43	-3%	0,802	2,61 ± 0,43	0%	0.997	2,62 ± 0,43
**Pi**	**0.037**	0,68 ± 0,21	-13%	0,307	0,59 ± 0,17	-9%	0.599	0,54 ± 0,18
**PDE**	0.254	2,89 ± 0,58	5%	0,599	3,04 ± 0,49	-7%	0.259	2,83 ± 0,44
**PCr**	0.891	3,65 ± 0,62	-2%	0,893	3,58 ± 0,48	1%	0.984	3,60 ± 0,49
**ATP**	0.082	2,83 ± 0,49	-5%	0,693	2,70 ± 0,48	-7%	0.400	2,51 ± 0,48
**NAD**	–	0,38 ± 0,12	-8%	–	0.35 ± 0.14	3%	–	0.36 ± 0.11
**pH**	0.263	6,98 ± 0,03	0,0%	0,992	6,99 ± 0,02	-0,1%	0.343	6,98 ± 0,02
**Mg**	0.115	0,11 ± 0,02	-9%	0,148	0,10 ± 0,01	1%	0.923	0,10 ± 0,01

**r_TH**	**overall comparison of groups (multivariate)**<br>Statistics MANOVA: Wilks-Lambda p = **0.014**							
		**pairwise group comparison** (post-hoc Scheffé test)						
	omnibus	**fN (n=21)**	**Δmf**		**mN (n=26)**	**ΔCN**		**mC (n=40)**
	p-value	mean ± SD	rel. diff.	p-value	mean ± SD	rel. diff.	p-value	mean ± SD
**PME**	**0,010**	2.30 ± 0.33	-15%	**0.014**	1.96 ± 0.40	4%	0.680	2.05 ± 0.39
**Pi**	0,533	0.79 ± 0.19	-5%	0.825	0.75 ± 0.18	-3%	0.892	0.73 ± 0.24
**PDE**	0,790	2.61 ± 0.46	-2%	0.939	2.56 ± 0.36	3%	0.790	2.63 ± 0.39
**PCr**	**0,016**	3.70 ± 0.50	-13%	**0.018**	3.23 ± 0.52	5%	0.558	3.38 ± 0.60
**ATP**	**0,012**	2,43 ± 0,48	-11%	0,166	2,16 ± 0,44	-6%	0.586	2,03 ± 0,53
**NAD**	–	0.36 ± 0.10	3%	–	0.37 ± 0.10	0%	–	0.37 ± 0.11
**pH**	0,413	6.98 ± 0.02	0.2%	0.264	6.99 ± 0.02	0.0%	0.545	6.99 ± 0.03
**Mg**	0,157	0.10 ± 0.01	9%	0.163	0.11 ± 0.02	-5%	0.469	0.10 ± 0.02

**l_TH**	**overall comparison of groups (multivariate)**<br>Statistics MANOVA: Wilks-Lambda p = 0.359							
		**pairwise group comparison** (post-hoc Scheffé test)						
	omnibus	**fN (n=21)**	**Δmf**		**mN (n=26)**	**ΔCN**		**mC (n=40)**
	p-value	mean ± SD	rel. diff.	p-value	mean ± SD	rel. diff.	p-value	mean ± SD
**PME**	0,062	2,32 ± 0,31	-9%	0,151	2,10 ± 0,39	-1%	0.986	2,09 ± 0,40
**Pi**	0,643	0,84 ± 0,16	-5%	0,799	0,80 ± 0,16	-2%	0.976	0,78 ± 0,25
**PDE**	0,610	2,63 ± 0,40	-1%	0,976	2,60 ± 0,43	4%	0.943	2,70 ± 0,43
**PCr**	0,196	3,75 ± 0,53	-7%	0,335	3,50 ± 0,51	-1%	0.996	3,48 ± 0,64
**ATP**	0,078	2,45 ± 0,43	-10%	0,313	2,21 ± 0,46	-4%	0.773	2,12 ± 0,60
**NAD**	–	0.39 ± 0.14	-13%	–	0.34 ± 0.10	3%	–	0.35 ± 0.10
**pH**	0,746	6,99 ± 0,02	0,0%	0,755	6,99 ± 0,01	0,0%	0.992	6,99 ± 0,03
**Mg**	0,164	0,10 ± 0,01	10%	0,560	0,11 ± 0,01	-6%	0.146	0,10 ± 0,01

**r_BG**	**overall comparison of groups (multivariate)**<br>Statistics MANOVA: Wilks-Lambda p = 0.358							
		**pairwise group comparison** (post-hoc Scheffé test)						
	omnibus	**fN (n=21)**	**Δmf**		**mN (n=26)**	**ΔCN**		**mC (n=39)**
	p-value	mean ± SD	rel. diff.	p-value	mean ± SD	rel. diff.	p-value	mean ± SD
**PME**	0,888	2,17 ± 0,37	0%	0,995	2,16 ± 0,39	-2%	0.874	2,11 ± 0,31
**Pi**	0,050	0,61 ± 0,18	3%	0,982	0,63 ± 0,16	-15%	0.117	0,53 ± 0,19
**PDE**	0,894	2,66 ± 0,40	2%	**0,040**	2,70 ± 0,45	-2%	0.846	2,63 ± 0,42
**PCr**	0,287	3,48 ± 0,45	-7%	0,288	3,22 ± 0,54	2%	0.865	3,29 ± 0,53
**ATP**	**0,050**	2,49 ± 0,48	-5%	0,693	2,36 ± 0,40	-9%	0.233	2,15 ± 0,53
**NAD**	–	0.33 ± 0.10	6%	–	0.35 ± 0.11	6%	–	0.37 ± 0.10
**pH**	0,934	6,99 ± 0,03	0,0%	0,934	6,99 ± 0,02	0,0%	0.998	6,99 ± 0,02
**Mg**	0,914	0,11 ± 0,01	0%	0,946	0,11 ± 0,01	0%	0.991	0,11 ± 0,01

l_BG	overall comparison of groups (multivariate)<br>Statistics MANOVA: Wilks-Lambda p = 0.122													
		pairwise group comparison (post-hoc Scheffé test)												
	omnibus	fN (n=21)			Δmf		mN (n=26)			ΔCN		mC (n=40)		
	p-value	mean ± SD			rel. diff.	p-value	mean ± SD			rel. diff.	p-value	mean ± SD		
**PME**	0,379	2,41	±	0,41	-5%	0,520	2,30	±	0,30	-1%	0.985	2,29	±	0,34
**Pi**	0,397	0,72	±	0,21	-4%	1,000	0,69	±	0,19	-9%	0.488	0,63	±	0,21
**PDE**	0,660	2,42	±	0,39	5%	0,707	2,54	±	0,44	-3%	0.698	2,46	±	0,41
**PCr**	**0,014**	3,68	±	0,41	5%	0,306	3,88	±	0,53	-10%	**0.015**	3,50	±	0,55
**ATP**	0,091	2,53	±	0,47	-8%	0,315	2,32	±	0,38	-4%	0.766	2,23	±	0,54
**NAD**	–	0.33	±	0.10	15%	–	0.38	±	0.13	-18%	–	0.31	±	0.10
**pH**	0,566	7,00	±	0,03	0,0%	0,987	7,00	±	0,03	-0,1%	0.710	6,99	±	0,03
**Mg**	0,890	0,11	±	0,01	0%	0,900	0,11	±	0,02	0%	0.995	0,11	±	0,02

r_TL	overall comparison of groups (multivariate)<br>Statistics MANOVA: Wilks-Lambda p = **0.034**													
		pairwise group comparison (post-hoc Scheffé test)												
	omnibus	fN (n=21)			Δmf		mN (n=26)			ΔCN		mC (n=40)		
	p-value	mean ± SD			rel. diff.	p-value	mean ± SD			rel. diff.	p-value	mean ± SD		
**PME**	0,150	2,04	±	0,39	7%	0,484	2,18	±	0,47	5%	0.764	2,25	±	0,38
**Pi**	0,277	0,59	±	0,18	-2%	0,866	0,58	±	0,20	-9%	0.453	0,53	±	0,14
**PDE**	0,102	2,03	±	0,54	16%	0,088	2,36	±	0,52	-7%	0.487	2,20	±	0,51
**PCr**	0,338	3,50	±	0,63	8%	0,460	3,78	±	0,91	0%	1,000	3,79	±	0,71
**ATP**	0,138	2,30	±	0,47	-3%	0,873	2,23	±	0,46	-7%	0.367	2,07	±	0,46
**NAD**	–	0.25	±	0.10	28%	–	0.32	±	0.11	6%	–	0.34	±	0.11
**pH**	0,209	7,00	±	0,03	0,0%	0,999	7,00	±	0,03	-0,1%	0.420	6,99	±	0,02
**Mg**	0,342	0,12	±	0,02	-8%	0,688	0,11	±	0,02	-2%	0.854	0,11	±	0,02

l_TL	overall comparison of groups (multivariate)<br>Statistics MANOVA: Wilks-Lambda p = 0.579													
		pairwise group comparison (post-hoc Scheffé test)												
	omnibus	fN (n=21)			Δmf		mN (n=26)			ΔCN		mC (n=40)		
	p-value	mean ± SD			rel. diff.	p-value	mean ± SD			rel. diff.	p-value	mean ± SD		
**PME**	0,526	2,13	±	0,40	0%	1,000	2,13	±	0,49	4%	0.631	2,23	±	0,31
**Pi**	0,363	0,63	±	0,20	6%	0,819	0,67	±	0,26	-12%	0.366	0,59	±	0,20
**PDE**	0,516	1,90	±	0,39	4%	0,798	1,98	±	0,38	-6%	0.518	1,86	±	0,43
**PCr**	0,449	3,62	±	0,60	8%	0,449	3,91	±	0,81	-3%	0.792	3,78	±	0,82
**ATP**	0,662	2,27	±	0,43	-5%	0,734	2,15	±	0,47	-3%	0.870	2,10	±	0,52
**NAD**	–	0,29	±	0,11	10%	–	0,32	±	0,10	-9%	–	0,29	±	0,09
**pH**	0,815	7,00	±	0,03	0,0%	0,844	7,00	±	0,03	0,0%	0.987	7,00	±	0,03
**Mg**	0,903	0,11	±	0,02	0%	0,870	0,11	±	0,01	0%	0.976	0,11	±	0,02

r_FWM	overall comparison of groups (multivariate) Statistics MANOVA: Wilks-Lambda p = 0.216							
		**pairwise group comparison** (post-hoc Scheffé test)						
	omnibus	**fN (n=19)**	**Δmf**		**mN (n=25)**	**ΔCN**		**mC (n=37)**
	p-value	mean ± SD	rel. diff.	p-value	mean ± SD	rel. diff.	p-value	mean ± SD
**PME**	0,798	2,27 ± 0,32	4%	0,751	2,35 ± 0,41	-2%	0.894	2,30 ± 0,38
**Pi**	0,111	0,61 ± 0,21	3%	0,982	0,63 ± 0,18	-15%	0.145	0,54 ± 0,19
**PDE**	0,116	2,56 ± 0,50	5%	0,797	2,69 ± 0,53	-9%	0.118	2,44 ± 0,41
**PCr**	0,904	3,40 ± 0,57	2%	1,000	3,46 ± 0,52	-1%	0.941	3,42 ± 0,44
**ATP**	**0,024**	2,60 ± 0,43	-5%	0,676	2,47 ± 0,45	-8%	0.199	2,27 ± 0,49
**NAD**	–	0,30 ± 0,13	17%	–	0,35 ± 0,15	-11%	–	0,31 ± 0,10
**pH**	0,725	6,98 ± 0,02	0,1%	0,658	6,99 ± 0,02	-0,1%	0.738	6,98 ± 0,03
**Mg**	0,698	0,11 ± 0,01	0%	0,899	0,11 ± 0,01	0%	0.970	0,11 ± 0,01

l_FWM	overall comparison of groups (multivariate) Statistics MANOVA: Wilks-Lambda p = 0.086							
		**pairwise group comparison** (post-hoc Scheffé test)						
	omnibus	**fN (n=19)**	**Δmf**		**mN (n=26)**	**ΔCN**		**mC (n=38)**
	p-value	mean ± SD	rel. diff.	p-value	mean ± SD	rel. diff.	p-value	mean ± SD
**PME**	0,931	2,22 ± 0,33	0%	0,993	2,23 ± 0,43	-2%	0.934	2,19 ± 0,34
**Pi**	0,198	0,66 ± 0,24	-9%	0,661	0,60 ± 0,21	-8%	0.666	0,56 ± 0,17
**PDE**	0,386	2,14 ± 0,51	6%	0,592	2,27 ± 0,30	1%	0.958	2,30 ± 0,36
**PCr**	**0,018**	3,33 ± 0,38	12%	**0,025**	3,73 ± 0,45	-7%	0.112	3,47 ± 0,52
**ATP**	0,058	2,35 ± 0,43	-2%	0,955	2,30 ± 0,37	-10%	0.158	2,08 ± 0,54
**NAD**	–	0.29 ± 0.10	6%	–	0.31 ± 0.13	3%	–	0.32 ± 0.11
**pH**	0,833	7,00 ± 0,04	0,0%	0,924	7,00 ± 0,03	-0,1%	0.839	7,00 ± 0,05
**Mg**	0,406	0,11 ± 0,02	0%	0,677	0,11 ± 0,02	-2%	0.909	0,11 ± 0,02

Absolute mean concentration values ± standard deviation of PME, Pi, PDE, PCr, ATP, NAD, and Mg are given as mmol/kg brain tissue.

Left and right half of the FGM voxel were not evaluated separately.

Δmf indicates the relative difference of metabolite values between fN and mN: $\Delta \mathrm{mf} = \frac{(\mathrm{mN}-\mathrm{fN})}{\mathrm{fN}}$.
ΔCN indicates the relative difference of metabolite values between mN and mC: $\Delta \mathrm{CN} = \frac{(\mathrm{mC}-\mathrm{m}\ \ )}{\mathrm{mN}}$.

The Wilks-Lambda test reflects the overall effect of the three groups on all seven metabolite values included in the MANOVA. NAD was excluded because of too many low-quality data. Post-hoc Scheffé test was used for paired comparison of groups.

p-values < 0.05 are marked in bold. *Values that remained significant after multiple comparisons correction.

Suppl_Fig9a: Results of Phosphorus MRS: concentration values - box plot

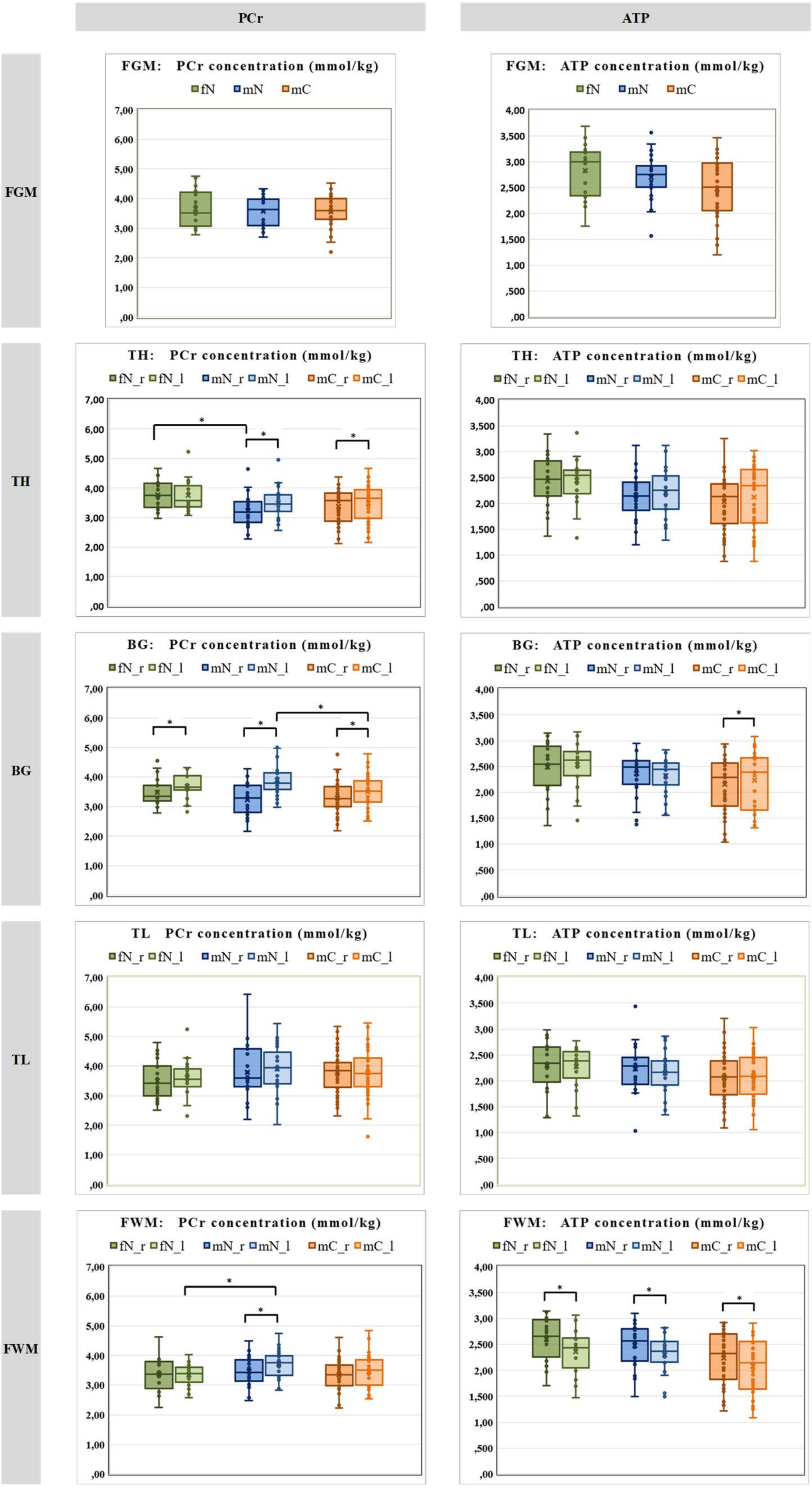

Box plots describing the variation of absolute metabolite concentration values of PCr and ATP in four different regions of the brain. Asterisk marks significant differences ($p<0.05$).

Suppl_Fig9b: Results of Phosphorus MRS: concentration values - box plot

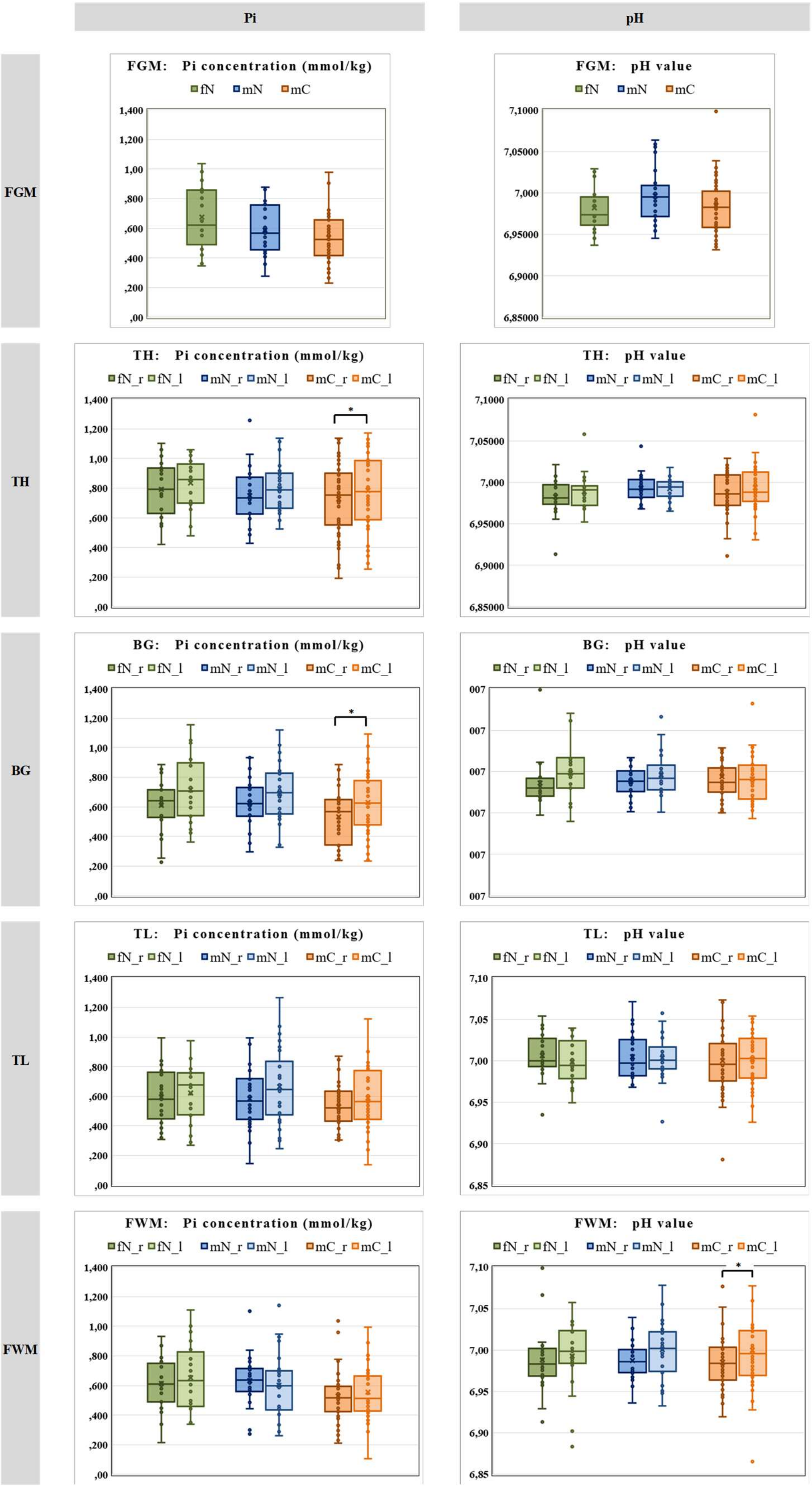

Box plots describing the variation of absolute metabolite concentration values of Pi and pH value in four different regions of the brain. Asterisk marks significant differences ($p<0.05$).

Suppl_Fig9c: Results of Phosphorus MRS: concentration values - box plot

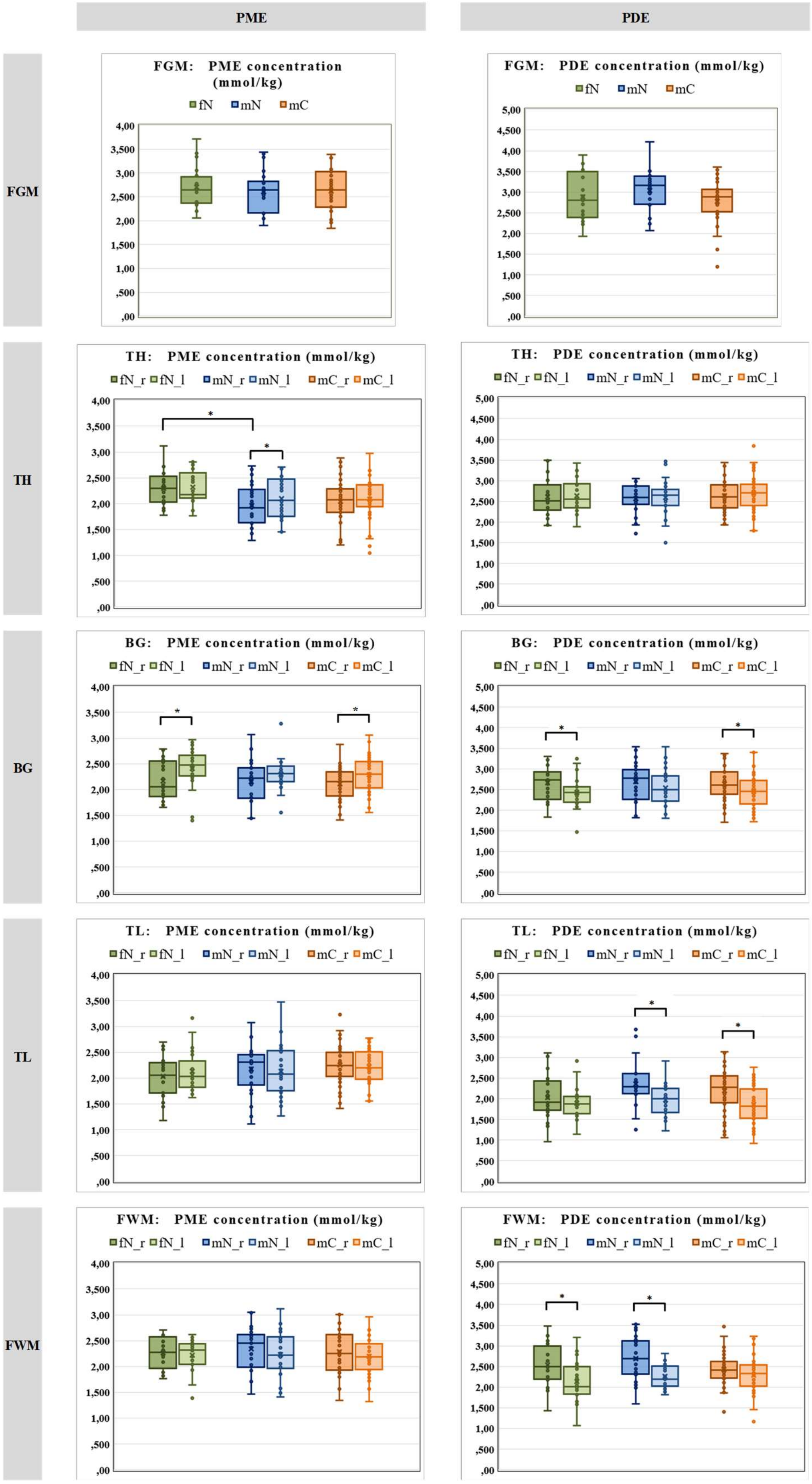

Boxplots describing the variation of absolute metabolite concentration values of PME and PDE in four different regions of the brain. Asterisk marks significant differences (p<0.05).

Suppl_Fig9d: Results of Phosphorus MRS: concentration values - box plot

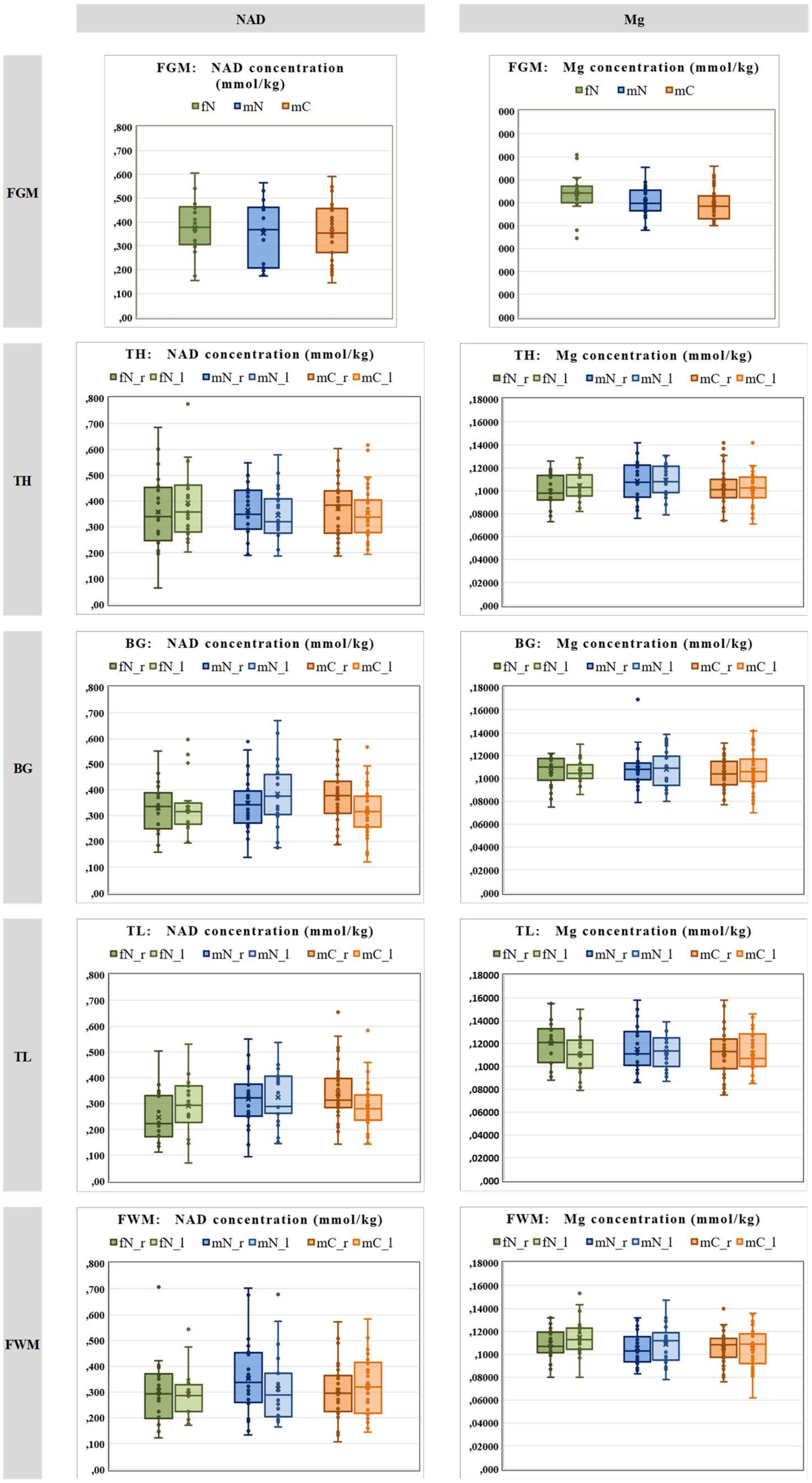

Boxplots describing the variation of absolute metabolite concentration values of NAD and Mg in four different regions of the brain. Asterisk marks significant differences ($p<0.05$).
