# Supplementary material for: Impact of cannabis use on brain metabolism using 31P and 1H magnetic resonance spectroscopy
Source: Neuroradiology. 2023 Sep 22;65(11):1631–48. doi: 10.1007/s00234-023-03220-y (PMC10567915; doi:10.1007/s00234-023-03220-y)
Supplement: Supplementary file 5 — (PDF 1.33 MB) [file 234_2023_3220_MOESM5_ESM.pdf]

Impact of cannabis use on brain metabolism using ^{31}P and ^{1}H magnetic resonance spectroscopy

Maximilian Fenzl[1] (ORCID 0000-0003-1011-2754) · Martin Backens[1] (ORCID 0000-0002-3414-696X) · Silviu Bodea[2] · Miriam Wittemann[3] · Florian Werler[4] · Jule Brielmaier[5] · Robert Christian Wolf[4] (ORCID 0000-0002-5358-5212) ·Wolfgang Reith[1]

1. Institute of Neuroradiology, Saarland University, 66421 Homburg, Germany
2. Helmholtz Zentrum Munich, German Research Center for Environmental Health Institute of Biological and Medical Imaging, 85748 Munich, Germany
3. Department of Psychiatry and Psychotherapy, Saarland University, 66421 Homburg, Germany
4. Department of General Psychiatry at the Center for Psychosocial Medicine, Heidelberg University, 69115 Heidelberg, Germany
5. Department of Obstetrics and Gynecology, RKH Clinic Ludwigsburg, 71640 Ludwigsburg, Germany

Suppl_Table6: T1 and T2 relaxation for Proton MRS

T1 (ms)	NAA	Cr	Cho	mI	Glx	Brain water	CSF
WM	1400	1300	1100	1000	1100	1000	4000
GM	1500	1400	1300	1000	1100	1500	4000

T2 (ms)	NAA	Cr	Cho	mI	Glx	Brain water	CSF
WM	280	150	200	200	180	75	1500
GM	250	150	230	200	180	95	1500

T1 and T2 values used for relaxation correction of proton metabolite values

Suppl_Table7: Results of 1H MRS - Comparison between groups

**FGM**	**overall comparison of groups (multivariate)** Statistics MANOVA: Wilks-Lambda: p = 0.047							
		**pairwise group comparison** (post-hoc Scheffé test)						
	omnibus	**fN (n=21)**	**Δmf**		**mN (n=26)**	**ΔCN**		**mC (n=40)**
	p-value	mean ± SD	rel. diff.	p-value	mean ± SD	rel. diff.	p-value	mean ± SD
**tNAA**	0.728	22.6 ± 3.5	1%	0.990	22.7 ± 3.1	-3%	0.759	22.1 ± 3.0
**tCr**	0.484	15.5 ± 2.4	4%	0.651	16.1 ± 2.8	1%	0.984	16.2 ± 1.8
**tCho**	**0.029**	4.1 ± 0.8	12%	0.057	4.6 ± 0.8	-1%	0.973	4.5 ± 0.5

**r_TH**	**overall comparison of groups (multivariate)** Statistics MANOVA: Wilks-Lambda: p = 0.455							
		**pairwise group comparison** (post-hoc Scheffé test)						
	omnibus	**fN (n=20)**	**Δmf**		**mN (n=25)**	**ΔCN**		**mC (n=39)**
	p-value	mean ± SD	rel. diff.	p-value	mean ± SD	rel. diff.	p-value	mean ± SD
**tNAA**	0.495	13.5 ± 1.9	2%	0.886	13.7 ± 2.3	-4%	0.500	13.1 ± 1.8
**tCr**	0.450	7.9 ± 1.3	5%	0.459	8.3 ± 1.1	-3%	0.712	8.1 ± 1.0
**tCho**	0.204	2.4 ± 0.3	8%	0.205	2.6 ± 0.4	-3%	0.610	2.5 ± 0.3

**r_TL**	**overall comparison of groups (multivariate)** Statistics MANOVA: Wilks-Lambda: p = 0.480							
		**pairwise group comparison** (post-hoc Scheffé test)						
	omnibus	**fN (n=20)**	**Δmf**		**mN (n=25)**	**ΔCN**		**mC (n=39)**
	p-value	mean ± SD	rel. diff.	p-value	mean ± SD	rel. diff.	p-value	mean ± SD
**tNAA**	0.508	15.8 ± 3.1	-3%	0.853	15.4 ± 2.9	-3%	0.846	15.0 ± 2.3
**tCr**	0.947	10.0 ± 2.0	-1%	0.972	9.9 ± 1.9	2%	0.950	10.0 ± 1.9
**tCho**	0.606	2.9 ± 0.7	7%	0.640	3.1 ± 0.6	-1%	0.978	3.0 ± 0.7

**r_FWM**	**overall comparison of groups (multivariate)** Statistics MANOVA: Wilks-Lambda: p = 0.593							
		**pairwise group comparison** (post-hoc Scheffé test)						
	omnibus	**fN (n=19)**	**Δmf**		**mN (n=23)**	**ΔCN**		**mC (n=39)**
	p-value	mean ± SD	rel. diff.	p-value	mean ± SD	rel. diff.	p-value	mean ± SD
**tNAA**	0.244	14.7 ± 1.6	4%	0.451	15.3 ± 1.7	-4%	0.272	14.7 ± 1.2
**tCr**	0.385	8.2 ± 1.0	5%	0.480	8.6 ± 1.1	0%	0.997	8.5 ± 1.0
**tCho**	0.791	2.7 ± 0.5	3%	0.795	2.8 ± 0.4	-1%	0.962	2.8 ± 0.4

Absolute mean concentration values ± standard deviation of tNAA, tCr and tCho are given as mmol/kg brain tissue.

Δmf indicates the relative difference of metabolite values between fN and mN: $\Delta\text{mf} = \frac{(\text{mN}- \quad)}{\text{fN}}$.
ΔCN indicates the relative difference of metabolite values between mN and mC: $\Delta\text{CN} = \frac{(\text{mC}-\text{m} \quad)}{\text{mN}}$.

The Wilks-Lambda test reflects the overall effect of the three groups on all three metabolite values included in the MANOVA. Post-hoc Scheffé test was used for paired comparison of groups.

p-values < 0.05 are marked in bold.

Suppl_Fig10a: Results of Proton MRS: concentration values - box plot

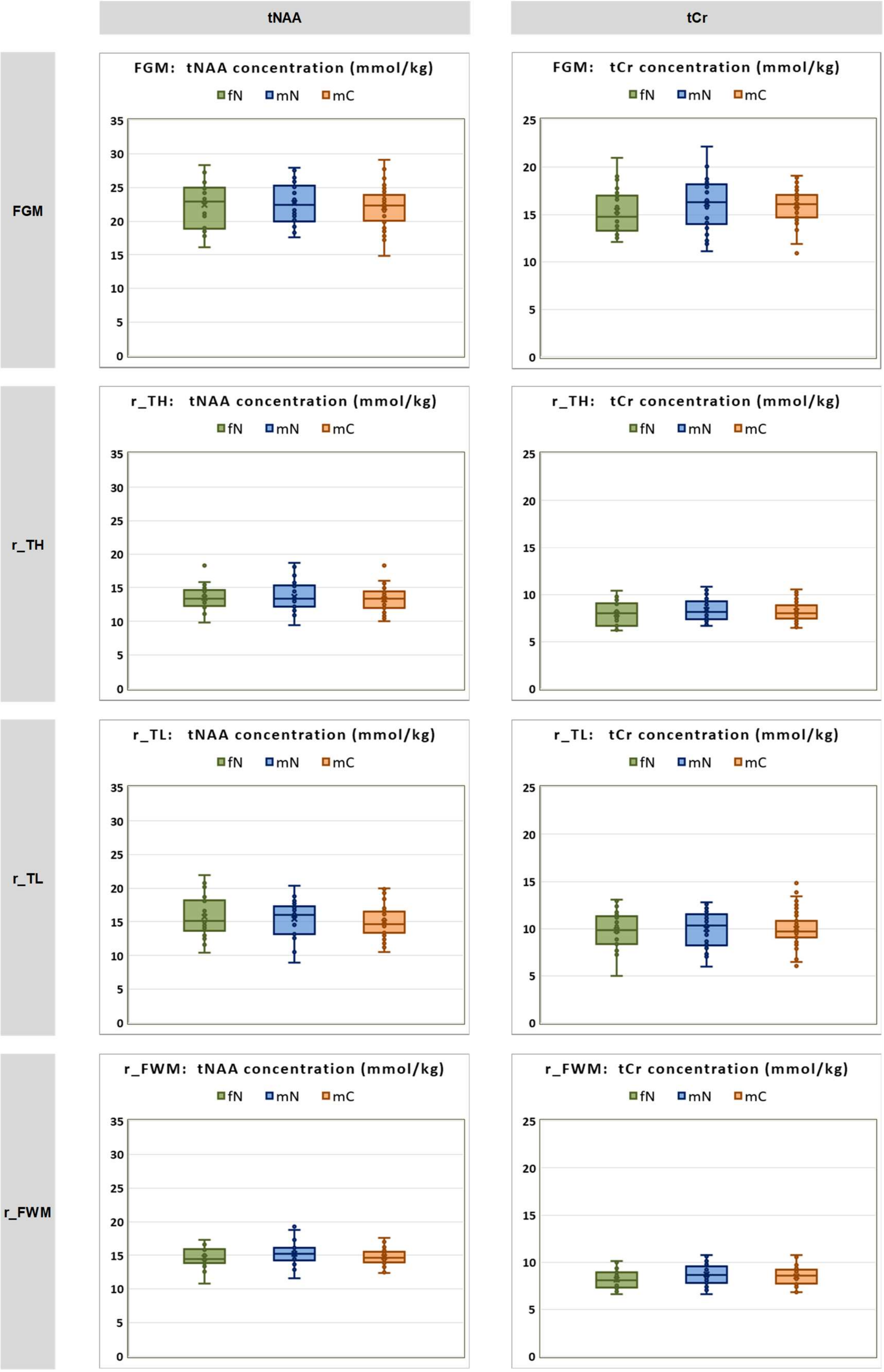

Box plots describing the variation of absolute metabolite concentration values of tNAA and tCr in four different regions of the brain. Asterisk marks significant differences (p<0.05).

Suppl_Fig10b: Results of Proton MRS: concentration values - box plot

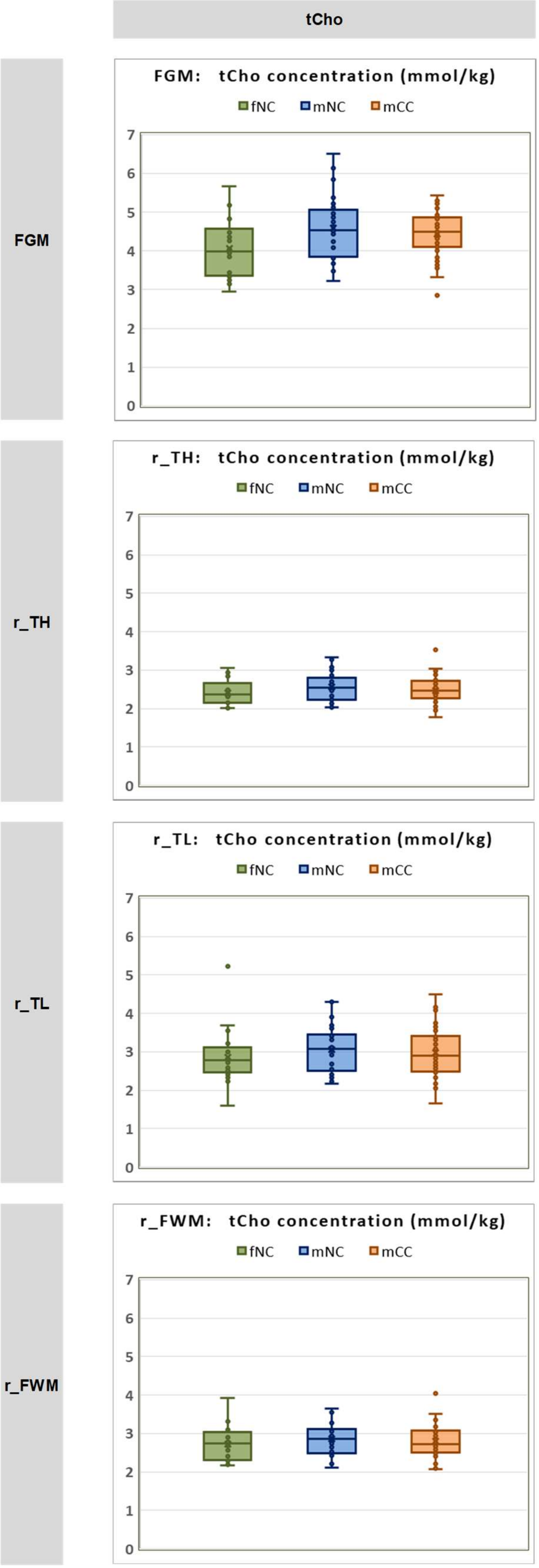

Box plots describing the variation of absolute metabolite concentration values of tCho in four different regions of the brain. Asterisk marks significant differences ($p<0.05$).

Suppl_Table8: Calculated ratio of concentrations PCr/Cr

PCr/Cr	pairwise group comparison (t-test)													
	fN			Δmf		mN			ΔCN		mC			
	mean ± SD			rel. diff.	p-value	mean ± SD			rel. diff.	p-value	mean ± SD			
**FGM**	0.32	±	0.09	-3%	0.695	0.31	±	0.10	-6%	0.451	0.29	±	0.06	
**r_TH**	1.01	±	0.47	-32%	**0.010**	0.68	±	0.28	16%	0.160	0.79	±	0.33	
BG	-		-			-		-	-	-	-		-	
**r_TL**	0.79	±	0.23	15%	0.362	0.90	±	0.57	-6%	0.678	0.85	±	0.35	
**r_FWM**	0.73	±	0.19	-3%	0.696	0.70	±	0.21	-3%	0.723	0.68	±	0.19	

Ratio of PCr and Cr concentrations. Cr concentration was obtained by: [Cr] = [tCr] –[PCr].
